# Symmetry-mode analysis for intuitive observation of structure–property relationships in the lead-free antiferroelectric (1−*x*)AgNbO_3_–*x*LiTaO_3_

**DOI:** 10.1107/S2052252519007711

**Published:** 2019-06-21

**Authors:** Teng Lu, Ye Tian, Andrew Studer, Narendirakumar Narayanan, Qian Li, Ray Withers, Li Jin, Y. Mendez-González, A. Peláiz-Barranco, Dehong Yu, Garry J. McIntyre, Zhuo Xu, Xiaoyong Wei, Haixue Yan, Yun Liu

**Affiliations:** a Research School of Chemistry, Australian National University, Canberra, ACT 2601, Australia; b Electronic Materials Research Laboratory, Xi’an Jiaotong University, Xi’an, Shannxi 710049, People’s Republic of China; c School of Engineering and Materials Science, Queen Mary University of London, London E1 4NS, UK; d Australian Nuclear Science and Technology Organisation, New Illawarra Road, Lucas Heights, NSW 2234, Australia; e Advanced Photon Source, Argonne National Laboratory, Argonne, IL 60439, USA; f Physics Faculty, Institute of Science and Technology of Materials, Havana University, Cuba

**Keywords:** anti-ferroelectricity, phase transitions, symmetry-mode analysis, crystal engineering, inorganic materials, materials science, inorganic chemistry

## Abstract

Functional materials are of critical importance to electronic and smart devices. A deep understanding of the structure–property relationship is essential for designing new materials. In this work, instead of utilizing conventional atomic coordinates, a symmetry-mode approach is successfully used to conduct structure refinement of the neutron powder diffraction data of (1−*x*)AgNbO_3_–*x*LiTaO_3_ (0 ≤ *x* ≤ 0.09) ceramics. This provides rich structural information that not only clarifies the controversial symmetry assigned to pure AgNbO_3_ but also explains well the detailed structural evolution of (1−*x*)AgNbO_3_–*x*LiTaO_3_ (0 ≤ *x* ≤ 0.09) ceramics, and builds a comprehensive and straightforward relationship between structural distortion and electrical properties. It is concluded that there are four relatively large-amplitude major modes that dominate the distorted *Pmc*2_1_ structure of pure AgNbO_3_, namely a Λ3 antiferroelectric mode, a T4+ *a* ^−^ *a* ^−^ *c* ^0^ octahedral tilting mode, an H2 *a* ^0^ *a* ^0^ *c* ^+^/*a* ^0^ *a* ^0^ *c* ^−^ octahedral tilting mode and a Γ4− ferroelectric mode. The H2 and Λ3 modes become progressively inactive with increasing *x* and their destabilization is the driving force behind the composition-driven phase transition between the *Pmc*2_1_ and *R*3*c* phases. This structural variation is consistent with the trend observed in the measured temperature-dependent dielectric properties and polarization–electric field (*P*-*E*) hysteresis loops. The mode crystallography applied in this study provides a strategy for optimizing related properties by tuning the amplitudes of the corresponding modes in these novel AgNbO_3_-based (anti)ferroelectric materials.

## Abstract 2

Symmetry-mode analysis has been used to construct the direct linkage between structure and properties for (anti)ferroelectric materials.

## 1. Introduction   

Functional materials with (anti)ferroelectricity (AFE/FE) offer innumerable applications for sensors, actuators, memory and energy-storage devices (Liu *et al.*, 2015 ▸; Hao *et al.*, 2009 ▸; Setter *et al.*, 2006 ▸; Haertling, 1999 ▸; Damjanovic, 1998 ▸). Lead-containing materials such as Pb(Zr,Ti)O_3_, Pb(Zr,Sn,Ti)O_3_ and Pb(Mg_1/2_Nb_2/3_)O_3_–PbTiO_3_ have already been manufactured into commercial devices due to their excellent properties (Park & Shrout, 1997 ▸; Bellaiche & Vanderbilt, 1999 ▸; Guo *et al.*, 2000 ▸; Mirshekarloo *et al.*, 2010 ▸), but environmental concerns nowadays prompt investigations into lead-free alternatives (Saito *et al.*, 2004 ▸; Shrout & Zhang, 2007 ▸). Recently, AgNbO_3_ (AN) has attracted researchers’ attention as a novel lead-free AFE material. It is reported that the recoverable energy density of pure AN ceramics can reach 2.1 J cm^−3^. After substituting 20% Nb^5+^ with Ta^5+^, however, the recoverable energy density doubles to ∼4.2 J cm^−3^, the highest value achieved to date in lead-free AFE ceramics (Zhao *et al.*, 2017 ▸; Tian *et al.*, 2016 ▸). One of the important reasons enabling such a high energy density in AN is its ultrahigh field-induced polarization (∼52 µC cm^−2^), which strongly suggests that AN should remain as the basis material from which to develop lead-free alternatives with high piezoelectric performance (Fu *et al.*, 2007 ▸, 2008 ▸, 2009 ▸, 2011*a* ▸). Referring to the investigation carried out by Fu *et al.* (2008 ▸), Li^+^ doping can stabilize the ferroelectricity of AN. More importantly, the synthesized single-crystalline (Ag_0.914_Li_0.086_)NbO_3_ exhibits a relatively large piezoelectric coefficient with a higher Curie temperature (*T*_C_), making it a competitive candidate for new lead-free piezoelectrics.

Currently, a considerable amount of effort, especially into chemical modification, is being made to improve the energy storage and/or piezoelectric capabilities of AN-based systems (Tian *et al.*, 2017 ▸; Zhao *et al.*, 2018 ▸, 2016 ▸). Nonetheless, the underlying structure and structural evolution of such doped materials still remain ambiguous and controversial. From a structural point of view, many room-temperature FE and/or AFE structures exhibit at least one large-amplitude (primary) distortive mode, in addition to the fundamental FE (polar **q** = 0 mode) and/or AFE modes which are directly responsible for their FE and/or AFE properties, when compared with their typically higher-symmetry paraelectric phases (Dove, 1997 ▸; Stokes *et al.*, 1991 ▸). The traditional single soft-mode approach is unable to describe the complete structural distortion in such circumstances. In seeking to understand the competing structural instabilities underlying the behaviour of such FE and AFE phases, it is thus very useful to utilize a mode crystallography approach, whereby the primary and induced secondary modes of distortion are clearly identified via symmetry-mode decomposition (Perez-Mato *et al.*, 2010 ▸). In such an approach, the room-temperature structure is described in terms of an undistorted parent structure and various additional distortive modes. Each mode is then associated with a specific allowed modulation wavevector and irreducible representation (irrep), as well as the mode amplitude.

This work therefore introduces this methodology into the structure refinement of neutron diffraction data collected from pure AN and associated compounds for better understanding of the chemically induced structural evolution and property changes, and is laid out in three parts. In the first part, symmetry-mode decomposition is successfully applied to pure AN for both the non-polar *Pbcm* and polar *Pmc*2_1_ space groups. It provides new insight into these two controversial symmetries, the origin of which will be addressed below, in terms of distortive modes. In the second part we extend the application of symmetry-mode analysis to the newly synthesized (1−*x*)AgNbO_3_–*x*LiTaO_3_ material system (ANLT100*x* hereafter) to build a more precise correlation between the structure and electrical properties of the ANLT system. The latter are presented in the third part. The symmetry-mode decomposition approach shows the variation in the relative amplitudes of the different modes as a function of the LiTaO_3_ dopant level, thereby enabling a better understanding of the structure of AN itself and its phase-transition behaviour under chemical modification, by comparison with conventional Rietveld fractional coordinate refinements. We believe this work not only presents a systematic investigation of a new AN-based solid-solution system, but also illustrates the influence of composition on the distortive-mode amplitudes and thus on the relative properties. Such an approach can guide future work in enhancing the AFE or FE properties of AN-based materials.

## 2. Results and discussion   

### 2.1. Symmetry-mode decomposition of AgNbO_3_   

**Figure 1:**
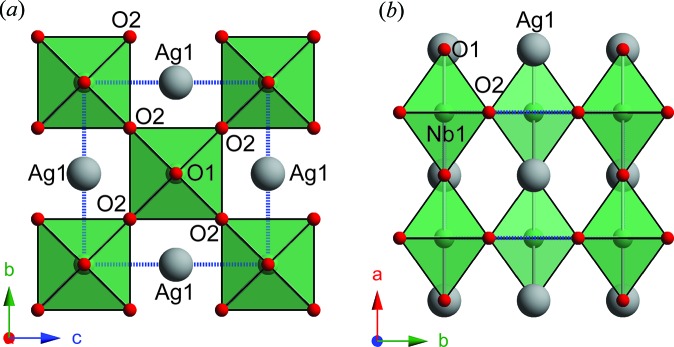
The parent *Ammm* structure viewed along (*a*) the *a* axis and (*b*) the *c* axis.

The average structure of AN at room temperature still remains controversial because either the *Pbcm* or the *Pmc*2_1_ space group can be used reasonably well for structure refinement based on X-ray and neutron powder diffraction data (Sciau *et al.*, 2004 ▸; Levin *et al.*, 2009 ▸; Yashima *et al.*, 2011 ▸). In this current work, the symmetry-mode decomposition approach is thus adopted to describe obvious differences between these two distorted structures proposed by Levin *et al.* (2009 ▸) and Yashima *et al.* (2011 ▸), respectively, to reveal a ‘hidden structural correlation’. Note that the *Pbcm* and *Pmc*2_1_ structures use different axes settings. In order to make them comparable and decomposed from the same parent structure, the *Pbcm* structure is transferred into a *Pmca* structure, based on the settings used by Yashima *et al.* (2011 ▸). The parent structure was then chosen as an undistorted *Ammm* structure (Fig. 1 ▸), which accommodates the octahedral rotation and avoids lattice strain. The unit-cell axes relationship between this *Ammm* structure (subscript A) and the pseudo-cubic perovskite structure with 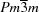 symmetry (subscript p) is **c**_p_ ≡ **a**_A_, **a**_p_ + **b**_p_ ≡ **b**_A_, −**a**_p_ + **b**_p_ ≡ **c**_A_.

Atomic displacements from the mode decomposition of the distorted *Pmca* and *Pmc*2_1_ structures are listed in the supporting information (Tables S1 and S2). The *Pmca* structure is the result of irrep distortions of the *Ammm* parent structure associated with five different irrep modes: Λ3, Y3−, Z2−, T4+ and H2. However, the associated atomic displacements for different modes display strong differences. Taking O3 as an example, the shift associated with the T4+ mode along the *a* axis is around 0.217 Å, while the shift resulting from the Z2− mode is only 0.004 Å, smaller than the standard deviation for the refinement. For the polar *Pmc*2_1_ structure, the origin is allowed to shift along the *c* direction. In this case, five more modes are allowed by comparison with the *Pmca* structure (Table S2), which are Γ4−, Λ1, Y2+, Z3+ and H4. Similarly, the atomic displacements associated with modes like Λ1 and Z2− are much smaller than the associated standard deviation. For each individual mode, the dimensions indicate the number of independent components or basis modes involved, and are larger for *Pmc*2_1_ (32) than for *Pmca* (15).

The global amplitude, *A*_τ_, is calculated by 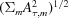, where *A*_τ,m_ denotes the amplitude for the specific component *m*. The dimensions and global amplitudes for each mode are listed together with the corresponding wavevectors **q** in Table 1 ▸ for both distorted structures. Clearly, the T4+, H2 and Λ3 modes have significantly larger global amplitudes in both cases. In the following, we identify the irrep modes whose condensation leads directly to the observed distortions. Referring to the ‘isotropy’ subgroups (Campbell *et al.*, 2006 ▸), the primary modes usually have the larger amplitudes. For *Pmca* symmetry, any two of the Λ3, T4+ and H2 modes could result in the observed distortions. Taking the relative amplitudes into consideration, we identify T4+ and H2 as the most important primary modes. Referring to the wavevectors listed in Table 1 ▸, the Λ3 mode can therefore be assigned to a secondary mode induced by the two most important co-existing primary T4+ and H2 modes, *i.e.* **q**_2_ = **q**_7_ − **q**_8_. However, for the lowering of the symmetry to *Pmc*2_1_, the condensation of the T4+ and H2 modes is insufficient. To obtain this structure another primary mode is required, namely the Γ4− mode at the zone centre with a relatively large amplitude. The *Pmc*2_1_ structure can then be considered as a subgroup of the *Pmca* structure. Therefore, we will focus on the four main modes T4+, H2, Λ3 and Γ4− step by step to understand the structural origin of the properties observed in silver niobate.

**Figure 2:**
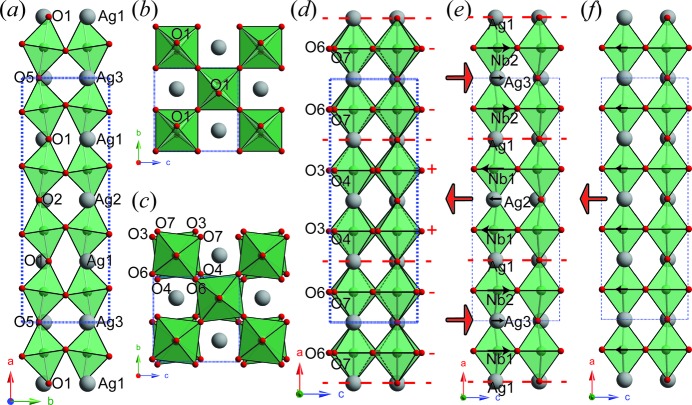
(*a*), (*b*) The distorted AN structure induced by the T4+ mode only, viewed along (*a*) the *c* axis and (*b*) the *a* axis. (*c*), (*d*) The distorted AN structure induced by the H2 mode only, viewed along (*c*) the *a* axis and (*d*) the *b* axis. The +/− signs on the right in panel (*d*) show the clockwise/anticlockwise rotation, respectively, of the right-hand column of octahedra around the *a* axis. (*e*) The distorted structure induced by the Λ3 mode only, and (*f*) that of the Γ4− mode only. The black arrows in panel (*e*) show the off-centre Nb^5+^ and Ag^+^ cation displacements, while the red arrows in panels (*e*) and (*f*) indicate the local spontaneous polarization. The horizontal dashed red lines represent antiphase boundaries for octahedral rotation around the *a* axis in panel (*d*) and cation displacements along the *c* axis in panel (*e*).

Figs. 2 ▸(*a*) and 2 ▸(*b*) show the distorted AN structure induced by the addition of the T4+ mode only to the *Ammm* parent structure. This **q**_7_ = [1/2 1 0]* T point mode occurs at the first Brillouin-zone boundary of its parent *Ammm* structure. The displacements involved correspond to a pure *R*(〈110〉_p_)-type octahedral rotation around the **c** = **c**_A_ ≡ −**a**_p_ + **b**_p_ axis, *i.e. a* ^−^ *a* ^−^ *c* ^0^ octahedral tilting in Glazer notation (Glazer, 1975 ▸). Figs. 2 ▸(*c*) and 2 ▸(*d*) show the distorted structure induced by the **q**_8_ = [1/4 1 0] (equivalent to [1/2 1/2 1/4]_p_*) H2 mode, which also occurs at the Brillouin-zone boundary and is also associated with octahedral rotation, but this time around the **a** = 4**a**_A_ = 4**c**_p_ axis. The H2 mode thus exhibits *R*(〈001〉_p_)-type octahedral rotation, *i.e.* rotation around the **a** axis, but not in the usual in-phase or antiphase rotation patterns expected for perovskites. If the structure is viewed along **a** [Fig. 2 ▸(*c*)], it can be seen that the NbO_6_ octahedra are antiphase tilted. In fact, this is because the adjacent NbO_6_ octahedra rotate alternately in a single column along the *a* axis [Fig. 2 ▸(*d*)]. If the ‘+’ sign denotes that the octahedron rotates clockwise and the ‘−’ sign denotes anticlockwise rotation viewed along **a**, the NbO_6_ octahedra [the right-hand column in Fig. 2 ▸(*d*)] rotate in the form of −−++−− around the *a* axis, or *a* ^0^ *a* ^0^ *c* ^+^/*a* ^0^ *a* ^0^ *c* ^−^. In other words, if the adjacent octahedra with in-phase tilt are regarded together as one unit, the dashed red lines in Fig. 2 ▸(*d*) can be considered as antiphase boundaries between these units. When combined with the T4+ mode, the resultant distorted structure is an *a* ^−^ *a* ^−^ *c* ^+^/*a* ^−^ *a* ^−^ *c* ^−^ tilting system, close to the reported *a* ^−^ *b* ^−^ *c* ^+^/*a* ^−^ *b* ^−^ *c* ^−^. In fact, Yashima *et al.* (2011 ▸) suggested equal tilting angles along [100]_p_ and [010]_p_, *i.e.* *a* ^−^ *b* ^−^ *c* ^+^/*a* ^−^ *b* ^−^ *c* ^−^ = *a* ^−^ *a* ^−^ *c* ^+^/*a* ^−^ *a* ^−^ *c* ^−^.

The T4+ and H2 modes together construct the overall octahedral tilting system in AN. It is worth noting that the octahedral tilting involves oxygen displacements and the mode amplitude is given in ångströms. The larger amplitude corresponds to a larger distortion. Furthermore, we will also include the tilting angles separately from the mode amplitude to describe the octahedral tilting fully. As mentioned above, the primary T4+ and H2 modes can directly produce a resultant *Pmca* structure.

The distortive structure induced by the next-strongest (induced secondary) Λ3 mode is shown in Fig. 2 ▸(*e*) and is mainly related to cation atomic displacements. Within one unit [two octahedral layers thick, shown between two dashed red lines in Fig. 2 ▸(*e*)], the Nb2 and Ag3 atoms are displaced off-centre along **c** [as shown by the black arrows in Fig. 2 ▸(*e*)], while anions such as O6, O7 and O5 are displaced to a lesser extent in the opposite direction. In this two-layer unit, the displaced ions would generate spontaneous polarization along +**c**, as shown by the red arrow. In the adjacent two-layer unit along **a**, cations such as Nb1 and Ag2 are displaced along −**c**, while O2, O3 and O4 again move in the opposite direction, forming an overall dipole moment along −**c**. Note that the Ag1 and O1 ions are located at the boundary between adjacent two-layer units and are thus not allowed to move along the *c* axis as a consequence of a required symmetry operation. Note that the dipole moment formed within each unit has the same magnitude, but the direction switches 180° from one unit to the next, resulting in an antiparallel dipole alignment. In other words, the Λ3 mode contributes directly to the observed antiferroelectricity in AN. Intriguingly, the two-layer units drawn in Figs. 2 ▸(*d*) and 2 ▸(*e*) show the same behaviour. After crossing each antiphase boundary, both the octahedral rotation around the *a* axis [in the case of Fig. 2 ▸(*d*)] and the dipole moment [in the case of Fig. 2 ▸(*e*)] change their sign. Considering Λ3 as an induced mode, it is evident that the antiferroelectric alignment in AN is very closely related to the observed *a* ^0^ *a* ^0^ *c* ^+^ */a* ^0^ *a* ^0^ *c* ^−^ (or ++−−) octahedral rotation pattern.

Finally, the distortive structure associated with the zone-centre Γ4− mode only, which, as a primary mode, differentiates the *Pmc*2_1_ structure from the *Pmca* structure by an additional ‘softening’, is shown in Fig. 2 ▸(*f*). For this ferroelectric **q** = 0 distorted structure, all ions move along the −**c** direction but with different magnitudes. For the cations, the displacements of Ag1, Ag2 and Ag3 (0.002 Å) are much smaller than those of Nb1 and Nb2 (0.059 Å). For the anions, the apical oxygens, *i.e.* O1, O2 and O5, are displaced by 0.025 Å, while the equatorial O3, O4, O6 and O7 anions are displaced by 0.027 Å. As a result, the spontaneous polarization points along −**c**. This Γ4− mode is therefore the origin of the weak ferroelectricity previously observed in silver niobate under a low electric field (*E* field) (Fu *et al.*, 2007 ▸). Undoubtedly, both the AFE Λ3 mode and the FE Γ4− mode respond to an externally applied *E* field, but the global amplitude of the FE mode is less than half that of the AFE mode. The competition between these two modes thus results in the observed ‘ferrielectricity’ (Yashima *et al.*, 2011 ▸), and also explains the appearance of a non-zero remnant polarization (*P*_r_) observed in the double *P*-*E* hysteresis loop of AN.

### 2.2. Symmetry-mode refinement   

In the previous section, we gave a detailed description of the condensation of the various symmetry modes, resulting in the two different space groups and structures reported for AN. In this section, we apply the above relationships to the ANLT100*x* series of samples for a systematic study of the variation in the modes by a mode-refinement procedure, which was conducted using the *FULLPROF* suite (Rodríguez-Carvajal, 1993 ▸) in conjunction with *ISODISTORT* (Campbell *et al.*, 2006 ▸). In contrast with conventional Rietveld refinement, the refinement of distortive modes enables a reasonable approach to refining the distorted structures, *e.g.* the primary modes should be refined first and modes with large amplitudes given higher priority. The reference structure was chosen to be the distorted perovskite-type structure with *Pmc*2_1_ space-group symmetry. This is due to the fact that both convergent-beam electron diffraction (CBED) and selected-area electron diffraction (SAED) prove the existence of a polar structure on the local scale (Tian *et al.*, 2016 ▸; Yashima *et al.*, 2011 ▸). Furthermore, as described in the previous section, the *Pmc*2_1_ structure exhibits an additional primary mode, the Γ4− mode, which explains the observed weak ferroelectricity in a low *E* field. It is thus more reasonable to investigate its variation as a function of LiTaO_3_ content. A systematic correlation of this mode with the ferroic properties may solve the apparent puzzle regarding the room-temperature AN structure.

**Figure 3:**
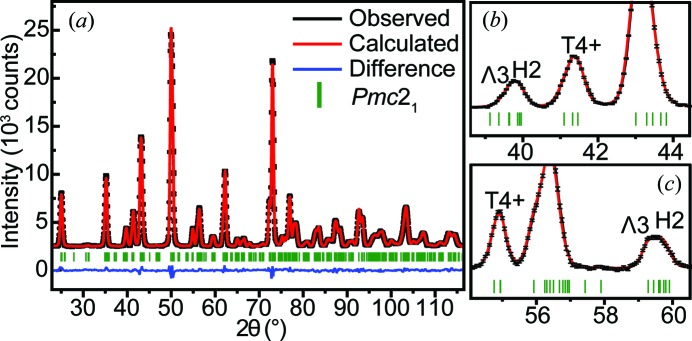
(*a*) Rietveld symmetry-mode refinement based on the *Pmc*2_1_ space group with the neutron powder diffraction (NPD) data of AN at room temperature. (*b*), (*c*) Selected reflections associated with the T4+, H2 and Λ3 modes.

Fig. 3 ▸(*a*) shows the neutron powder diffraction (NPD) data of pure AN collected in the 2θ range of 22–116°, while Figs. 3 ▸(*b*) and 3 ▸(*c*) show selected reflections associated with the T4+, H2 and Λ3 modes. It is clear that the symmetry-mode refinement approach provides detailed information about the reflection intensities associated with the different modes. For example, the reflections around 2θ = 41.5 and 55° are induced by the T4+ mode, related to the antiphase NbO_6_ octahedral rotation around [110]_p_. Additionally, the reflections around 39.8 and 59.5° can be attributed to the combination of the Λ3 and H2 modes, with their intensities mainly determined by the H2 mode as a result of its larger global amplitude.

**Figure 4:**
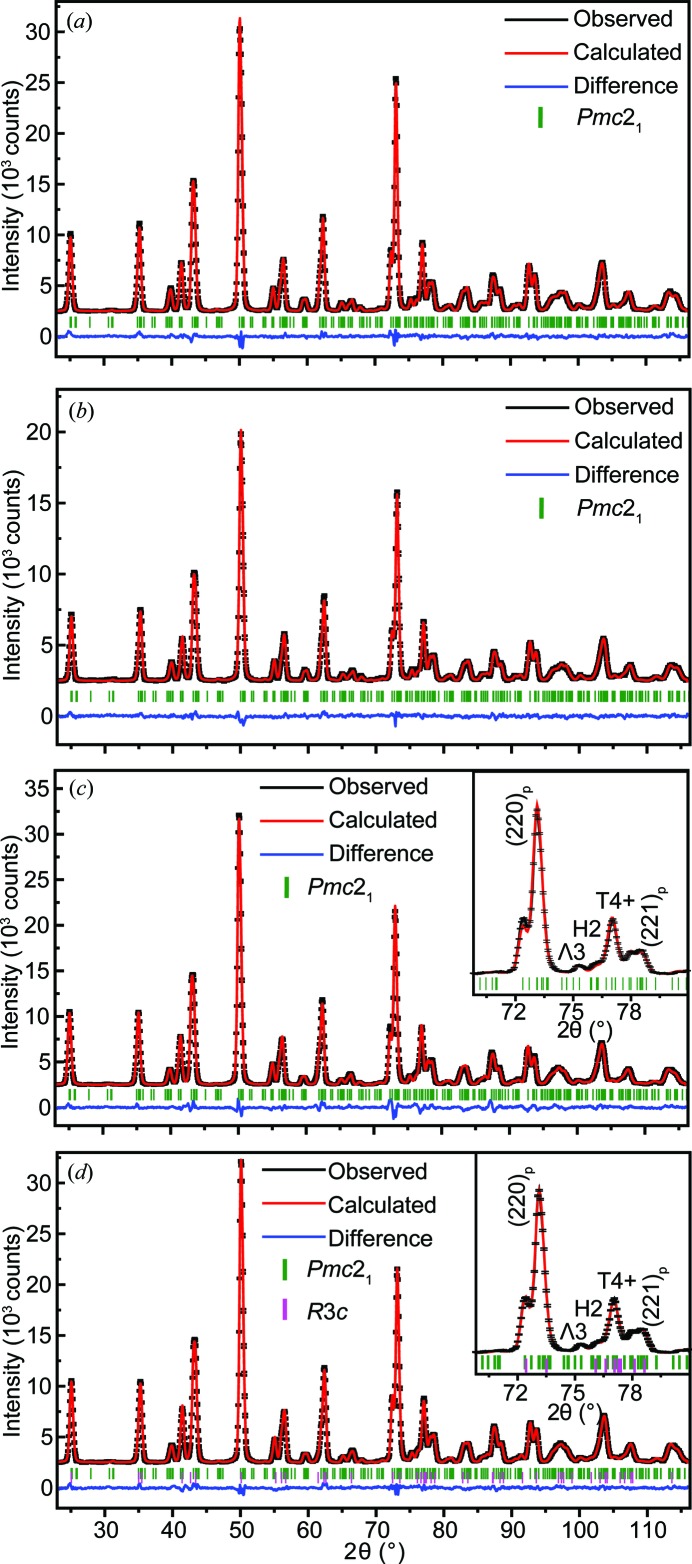
Rietveld symmetry-mode refinement of the NPD data of ANLT100*x*, (*a*) *x* = 0.03, (*b*) *x* = 0.045 and (*c*) *x* = 0.053 with a space group of *Pmc*2_1_, collected at room temperature. (*d*) Rietveld symmetry-mode refinement of the NPD pattern of ANLT5.3 in terms of the two-phase model (*R*3*c* + *Pmc*2_1_). The insert plots in panels (*c*) and (*d*) are enlargements of selected reflections, indexed by the related irrep modes.

The *Pmc*2_1_ single-phase model was also attempted on NPD patterns of other ANLT100*x* samples. Given the relatively low doping level (<10%), the Li^+^ and Ta^5+^ ions are fixed at the same positions of Ag^+^ and Nb^5+^, respectively. For samples with relatively small *x* values, *e.g. x* = 0.03 and 0.045, the *Pmc*2_1_ single-phase model leads to a reasonable refinement result [Figs. 4 ▸(*a*) and 4 ▸(*b*)]. However, for *x* = 0.053, the *Pmc*2_1_ single-phase model fails to fit the experimental data well [Fig. 4 ▸(*c*)] and a large divergence is especially observed in the 2θ range of 70 to 80°. The insert plot indicates that the selected peaks are poorly fitted. Referring to the pseudo-cubic perovskite structure (subscript p), it is found that the largest difference appears in calculating parent reflections such as 〈220〉_p_* and 〈221〉_p_*. For example, the intensity ratio of the split [220]_p_*/[202]_p_* reflections is incorrectly estimated. Furthermore, the intensity of the 1/2〈531〉_p_* reflection, determined by the amplitude of the T4+ mode, is underestimated. Interestingly, the calculated intensities of reflections associated with the Λ3 and H2 modes are in good agreement with the experimental data.

A bond-valence sum (BVS) calculation (Brown, 1981 ▸) suggests that the substitution of Ta^5+^ for Nb^5+^ does not make a big difference, but the replacement of an Li^+^ ion for an Ag^+^ ion would strongly destabilize the parent AN structure. Previous studies of Li-doped AgNbO_3_ systems (Fu *et al.*, 2008 ▸, 2011*b* ▸; Khan *et al.*, 2012 ▸) also reported that, with higher Li^+^ content, the average structure is transformed into a rhombohedral phase and the properties change accordingly. Intuitively, the features of the underestimated reflections in Fig. 4 ▸(*c*) are consistent with the patterns induced by the presence of an *R*3*c* symmetry structure. A two-phase model refinement (space groups *Pmc*2_1_ and *R*3*c*) was thus applied to the ANLT5.3 pattern, which evidently improved the refinement quality [Fig. 4 ▸(*d*)].

For the *R*3*c* phase, the symmetry-mode decomposition has been done with reference to the reported high-temperature cubic structure of AN [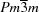 symmetry, ICSD (Inorganic Crystal Structure Database, http://www2.fiz-karlsruhe.de/icsd_home.html) refcode 55649] (Sciau *et al.*, 2004 ▸). The basis of this distorted structure is set as: **a**_r_ ≡ **a**_p_ + **c**_p_, **b**_r_ ≡ **b**_p_ − **c**_p_ and **c**_r_ ≡ −2**a**_p_ + 2**b**_p_ + 2**c**_p_. For the *R*3*c* structure, condensation of two primary modes with large amplitudes, namely Γ4− **q**_r0_ = [0 0 0]_p_* and R4+ **q**_r1_ = [1/2 1/2 1/2]_p_*, will lead to the observed distortions. The Γ4− **q**_r0_ = [0 0 0]_p_* mode, which allows off-centre ionic shifts along the *c* axis (the [111]_p_ direction), contributes to the FE spontaneous polarization. The R4+ **q**_r1_ = [1/2 1/2 1/2]_p_* mode, on the other hand, is associated with antiphase octahedral rotation around the [111]_p_ direction, *i.e.* *a* ^−^ *a* ^−^ *a* ^−^ octahedral tilting in Glazer notation (Glazer, 1975 ▸).

**Figure 5:**
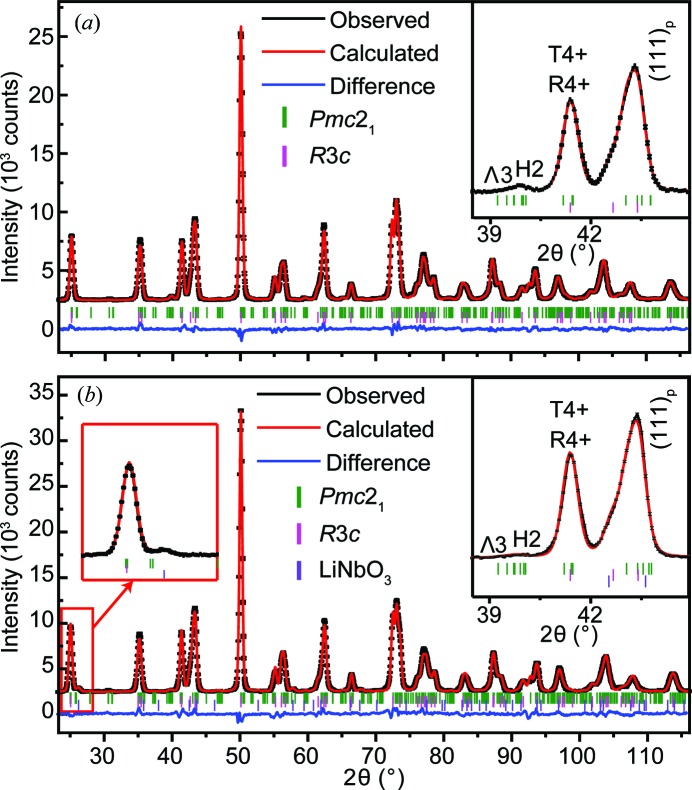
Plots of NPD data with Rietveld analysis for (*a*) ANLT6 and (*b*) ANLT9. The insert plots at the top right are enlargements of selected regions. The insert plot with a red frame on the left of panel (*b*) is an enlargement showing the presence of an impure LiNbO_3_ phase.

With further doping of LiTaO_3_, *i.e. x* = 0.06 and 0.09, the features associated with the *R*3*c* phase become more obvious. As shown in Fig. 5 ▸(*a*), the 〈111〉_p_* reflections contain a small shoulder at the lower 2θ angle which does not belong to the *Pmc*2_1_ phase. The two-phase model was also applied to refine the data of both ANLT6 and ANLT9 (Fig. 5 ▸), resulting in good agreement between the observed and calculated patterns. It should be noted that both the T4+ mode for the *Pmc*2_1_ phase and the R4+ mode for the *R*3*c* phase contribute to the intensities of the **G**_p_ ± [1/2 1/2 1/2]_p_* reflections, so both irrep notations are labelled. For *x* = 0.09, an additional peak observed at 26° [Fig. 5 ▸(*b*), labelled by the red rectangular symbol] is probably from an impure LiNbO_3_ phase as previously reported (Khan *et al.*, 2012 ▸). Note that due to overlapping, peak intensities are easily influenced by cross-talk. Furthermore, the BVS calculation indicates that Li^+^ would prefer to move away from the Ag site to an interstitial site (Alonso *et al.*, 2000 ▸; Brant *et al.*, 2012 ▸). Therefore, the reliability factors of the refinement on ANLT9 are not as good as those for the lower-level LiTaO_3_ doped samples (Table S3). Due to the detection of a secondary phase, a deviation in *x* from 9% for LiTaO_3_ is probably expected. The refinement results reveal that the molar fraction of this secondary phase is around 2.5%, suggesting that the dopant level of LiTaO_3_ is around 6.5%. Application of the modified composition did indeed improve the reliability factors (*R*_p_ changes from 0.0268 to 0.0260). Therefore, in the following we assume a LiTaO_3_ content of 6.5% for ANLT9 in order to guide the reader’s understanding of the structural evolution as a function of LiTaO_3_ content. Details of the refined atomic positions of the ANLT100*x* system are shown in the supporting information (Tables S4–S9).

**Figure 6:**
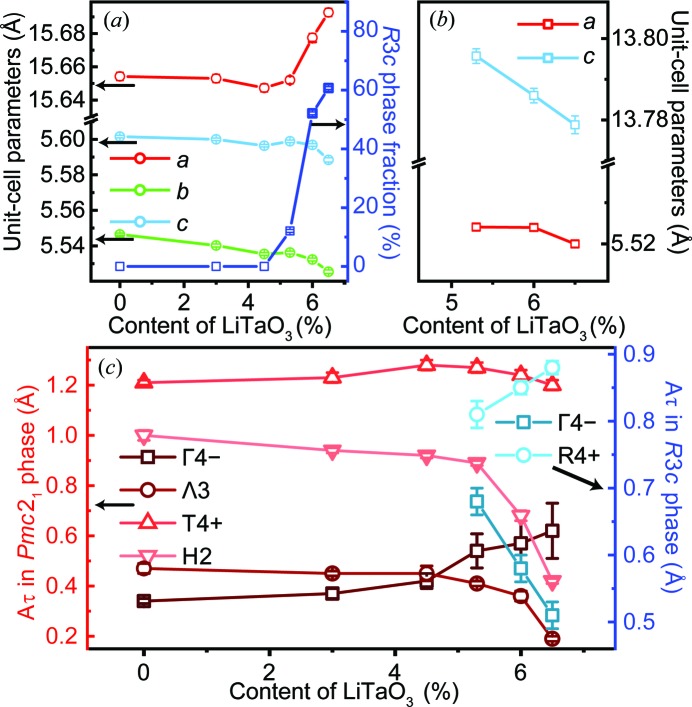
(*a*) Refined lattice parameters of the *Pmc*2_1_ phase and the phase fraction of the *R*3*c* phase. (*b*) Lattice parameters of the *R*3*c* phase. (*c*) The global amplitudes of the main modes in both the *Pmc*2_1_ and *R*3*c* phases, changing as a function of LiTaO_3_ content.

Fig. 6 ▸ shows the structural evolution of the ANLT100*x* materials as a function of LiTaO_3_ content. In the *Pmc*2_1_ single-phase region, *i.e. x* < 0.053, the unit-cell parameters (*a*, *b* and *c*) decrease gradually with respect to the *x* value [Fig. 6 ▸(*a*)]. This shrinkage of the unit cell is possibly due to the introduction of Li^+^, whose ionic radius is 92 pm compared with the 128 pm radius of Ag^+^ (Shannon, 1976 ▸). When the *R*3*c* phase appears (at *x* = 0.053), the *a*, *b* and *c* values for the ortho­rhombic phase exhibit a slight increase. In the two-phase region, *i.e. x* ≥ 0.053, with further doping the lattice parameter *a* increases, whereas *b* and *c* decrease. On the other hand, the fraction of the *R*3*c* phase increases from 12.1 to 53% for 0.053 ≤ *x* ≤ 0.06. For the *R*3*c* phase, both *a* and *c* are reduced with increasing *x* [Fig. 6 ▸(*b*)]. The variation in the unit-cell parameters of both the *Pmc*2_1_ and *R*3*c* structures cannot simply be explained by the ionic radii; it is probably linked to octahedral rotation and interaction between the two phases.

Fig. 6 ▸(*c*) shows the global amplitude (*A*_τ_) of the main modes changing as a function of LiTaO_3_ content for both the *Pmc*2_1_ and *R*3*c* phases. Larger *A*_τ_ values imply larger atomic displacements and a more highly distorted structure. In the *Pmc*2_1_ phase, referring to the mode decomposition in pure AN, the four critical modes can be divided into ionic displacements (FE Γ4− and AFE Λ3) and octahedral rotations (H2 and T4+). The amplitudes of the H2 and Λ3 modes decrease upon Li^+^ doping. However, the slope has an inflexion point at *x* = 0.053 and descends rapidly upon further Li^+^ doping. The amplitude of the FE Γ4− mode, on the other hand, displays the opposite trend to that of the AFE Λ3 mode. It is noteworthy that the deviation of the Γ4− mode’s amplitude becomes quite large when *x* ≥ 0.053, indicating that the variation of this parameter has less impact on the refinement results.

**Figure 7:**
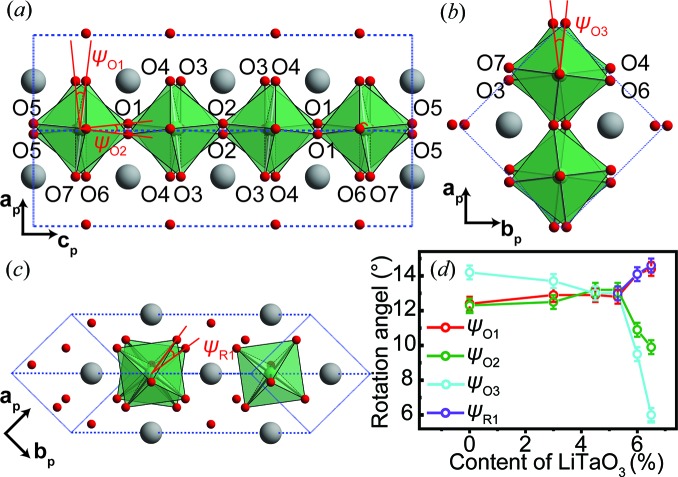
The distorted structure induced by (*a*) the T4+ mode for the *Pmc*2_1_ phase viewed along the *b*_p_ axis, (*b*) the H2 mode for the *Pmc*2_1_ phase viewed along the *c*_p_ axis and (*c*) the R4+ mode for the *R*3*c* phase viewed along the *c*_p_ axis. (*d*) The LiTaO_3_ content-dependent rotation angles.

In Section 2.1 we described the distorted structure induced by the largest-amplitude single modes, and also found that the overall octahedral tilting pattern is induced by a combination of T4+ and H2 modes. Instead of the related oxygen displace­ments, the tilting angles can also be used to reflect the degrees of distortion for these octahedral rotation modes. As shown in Fig. 7 ▸(*a*), the structure induced by the T4+ mode can be visually expressed by the tilting angles between the two adjacent NbO_6_ octahedra viewed along either [100]_p_ or [010]_p_. Here, Ψ_O1_ and Ψ_O2_ (subscript O denotes the orthorhombic phase) are used to illustrate the tilt angles. If there is no octahedral distortion, Ψ_O1_ = Ψ_O2_. The H2 mode is associated with the in- or antiphase rotation around [001]_p_ and Ψ_O3_ is used to characterize the tilting angle for this mode [Fig. 7 ▸(*b*)].

In the *R*3*c* phase, the R4+ mode denotes *a* ^−^ *a* ^−^ *a* ^−^ octahedral tilting, and therefore the tilt angle along any 〈100〉_p_ direction, Ψ_R1_ (subscript R denotes the rhombohedral phase), is used to describe this distorted structure [Fig. 7 ▸(*c*)]. Fig. 7 ▸(*d*) shows the quantitative analysis of these rotation angles. For Ψ_O1_ and Ψ_O2_, they are almost equivalent when *x* ≤ 0.053, and both increase slightly as the doping level increases. When *x* ≥ 0.053, Ψ_O1_ and Ψ_O2_ behave differently: Ψ_O1_ increases, whereas Ψ_O2_ decreases. This suggests that the octahedral distortion is accompanied by the appearance of the *R*3*c* phase. Furthermore, the decrease in Ψ_O2_ also explains the increase in the unit-cell parameter *a* for *x* ≥ 0.053. The tilt angle Ψ_R1_ increases with increases in the heavily underbonded Li^+^ dopants. For the H2 mode, Ψ_O3_ decreases slightly before the appearance of the *R*3*c* phase and then sharply when the *R*3*c* phase dominates the samples. This behaviour is very similar to that of primary modes that vary as a function of temperature in other materials (Khalyavin *et al.*, 2014 ▸; Faik *et al.*, 2012 ▸; Gómez-Pérez *et al.*, 2016 ▸). Therefore, the destabilization of the H2 mode is very important to this composition-driven phase transition in the ANLT100*x* system.

Note that the sudden drop in Ψ_O3_ is deduced by the change in the degree of ordering of *a* ^0^ *a* ^0^ *c* ^+^/*a* ^0^ *a* ^0^ *c* ^−^ octahedral tilting. This is probably due to the fact that the rotation around [001]_p_ creates differing periodicities or is totally disordered (Khan *et al.*, 2012 ▸; Wang *et al.*, 2011 ▸; Guo *et al.*, 2011 ▸; Liu *et al.*, 2012 ▸; Bellaiche & Íñiguez, 2013 ▸; Prosandeev *et al.*, 2013 ▸), *i.e.* the associated modulation wavevector moves along the H line in the first Brillouin zone of the parent *Ammm* structure. In the Li-doped AgNbO_3_ material system (Khan *et al.*, 2012 ▸, 2010 ▸), electron diffraction patterns show **G**_p_ ± [1/2 1/2 1/3]_p_* satellite reflections, which in turn indicate a movement to the zone boundary (T point) of the H2 mode. Finally, the combination of the T2+ (*a* ^0^ *a* ^0^ *c* ^−^) and T4+ modes induces the *a* ^−^ *a* ^−^ *a* ^−^ octahedral tilting observed in the *R*3*c* phase, and therefore the variation in the H2 mode locally builds an intermediate structure between the *Pmc*2_1_ and *R*3*c* phases.

As mentioned above, the Γ4− mode is responsible for ferroelectricity in both the *Pmc*2_1_ and *R*3*c* phases, while the Λ3 mode is associated with antiferroelectricity for the *Pmc*2_1_ phase. Note that the Λ3 mode can be regarded as a secondary mode induced by primary H2 and T4+ modes, and its composition-dependent amplitude follows the same trend as the H2 mode, suggesting an improper AFE nature of AN (Bellaiche & Íñiguez, 2013 ▸).

**Figure 8:**
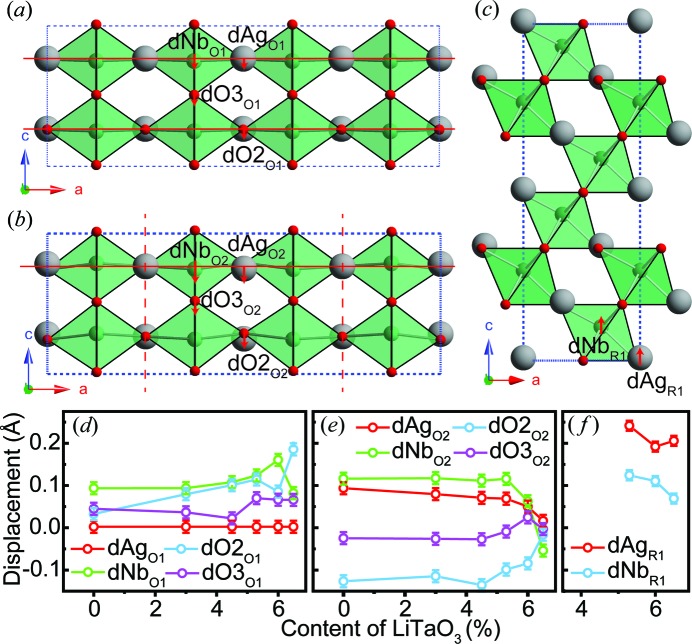
The distorted structure induced by (*a*) the Γ4− mode and (*b*) the Λ3 mode for the *Pmc*2_1_ phase, viewed along the *b* axis. The red solid lines indicate the *z* coordinates for the undistorted structure, the vertical dashed red lines in panel (*b*) show the boundaries of the two-layer octahedral units within which the dipole moments share the same direction, and the red arrows denote the displacements of the respective ions. (*c*) The distorted structure induced by the Γ4− mode for the *R*3*c* phase, viewed along the *b* axis. (*d*), (*e*), (*f*) The ionic displacements induced by (*d*) the Γ4− mode, (*e*) the Λ3 mode in the *Pmc*2_1_ phase and (*f*) the Γ4− mode in the *R*3*c* phase as a function of LiTaO_3_ content.

In order to analyse the (anti)ferroelectricity further, the ionic displacements associated with the different modes are extracted and plotted as a function of composition in Fig. 8 ▸. Although some atomic displacements along the *b* axis are involved in both Γ4− and Λ3 modes in the *Pmc*2_1_ phase (Table S2), the refined values are quite small. Furthermore, because the (anti)parallel dipole moments are aligned along the *c* axis, only displacements from the *z* coordinates are considered. For the Γ4− mode in the *Pmc*2_1_ phase [Fig. 8 ▸(*a*)], *d*O2_O1_ (displacement of the apical oxygen), *d*O3_O1_ (displacement of the equatorial oxygens), *d*Ag_O1_ (Ag/Li) and *d*Nb_O1_ (Nb/Ta) denote the ionic displacements along −**c** from the undistorted position (subscripts O and 1 indicate the ortho­rhombic phase and the Γ4− mode, respectively). Fig. 8 ▸(*b*) gives a schematic description of the ionic displacements associated with the Λ3 mode. As the dipole moments exhibit antiparallel alignment with the same amplitude, only atoms involved in one unit are extracted (subscript 2 denotes the Λ3 mode). In this case, only the displacements of Ag/Li2 (*d*Ag_O2_), Nb/Ta1 (*d*Nb_O2_), apical oxygen (*d*O2_O2_) and equatorial oxygen (*d*O3_O2_) are extracted. Similarly, *d*Ag_R1_ (Ag/Li) and *d*Nb_R1_ (Nb/Ta) are used to describe the ferroelectricity in the *R*3*c* phase [Fig. 8 ▸(*c*)]. Note that in the *R*3*c* phase, the *z* coordinate of the oxygen is fixed to zero, therefore cationic shifts are enough to describe the spontaneous polarization.

It is interesting that, even though the amplitude of the Γ4− mode in the *Pmc*2_1_ phase exhibits a systematic increase as a function of *x*, the change in both *d*O2_O1_ and *d*Nb_O1_ indicates that spontaneous polarization does not follow the same trend, especially when *x* ≥ 0.053 [Fig. 8 ▸(*d*)]. The zone-centre mode is very hard to calculate accurately via powder diffraction. The large deviations suggest that the structure models based on both *Pmc*2_1_ and *Pbcm* reproduce the experimental pattern reasonably well. By contrast, the refined values of *d*O2_O2_, *d*O3_O2_, *d*Ag_O2_ and *d*Nb_O2_ involved in the Λ3 mode change systematically as a function of LiTaO_3_ content [Fig. 8 ▸(*e*)]. Before introducing the LiTaO_3_, the cations and anions are displaced in opposite directions, *i.e.* *d*O2_O2_ and *d*O3_O2_ < 0, and *d*Ag_O2_ and *d*Nb_O2_ > 0, leading to a strong spontaneous polarization within any one two-octahedral-layer unit. With increasing *x*, the displacements of the anions and cations begin to converge, until a sudden change occurs at *x* ≃ 0.053. This behaviour suggests that the dipole moment in each sublattice becomes smaller, *i.e.* the antiferroelectricity is weakening. For *x* ≥ 0.053, the *R*3*c* phase emerges and its fractional content rises with further increase in *x*, whereas the diminishing *Pmc*2_1_ phase is simultaneously accompanied by a weakening antiferroelectricity. As a consequence, the ferroic properties are expected to be dominated by the *R*3*c* phase in this composition region. As shown in Fig. 8 ▸(*f*), for the sample with the largest *R*3*c* phase fraction (*x* ≥ 0.06), *d*Ag_R1_ remains unchanged while *d*Nb_R1_ shows a slight decrease.

### 2.3. Electrical properties   

**Figure 9:**
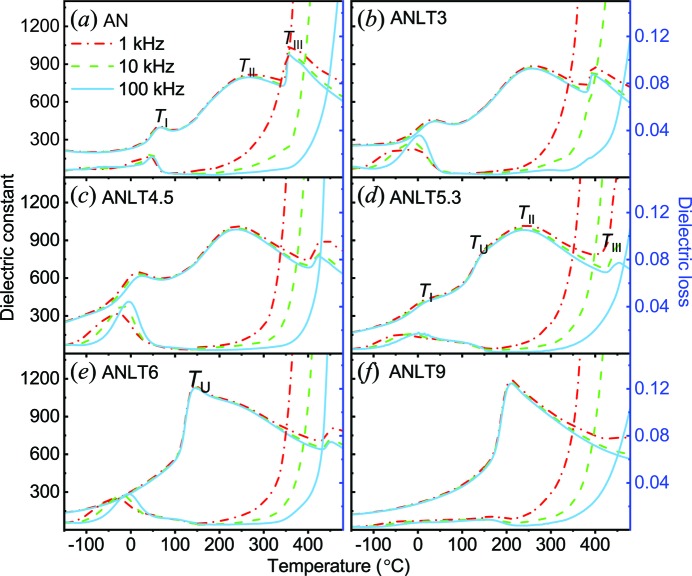
Temperature-dependent dielectric spectra for (*a*) AN, (*b*) ANLT3, (*c*) ANLT4.5, (*d*) ANLT5.3, (*e*) ANLT6 and (*f*) ANLT9 bulk ceramics.

Fig. 9 ▸ shows the temperature-dependent dielectric spectra of ANLT100*x* bulk ceramics. That for pure AN contains three evident dielectric constant peaks, *T*_I_ (∼70°C), *T*_II_ (∼270°C) and *T*_III_ (∼350°C), in the measured temperature range from −150 to 480°C, consistent with the previously reported experimental results (Fu *et al.*, 2007 ▸). In this temperature range, AN is reported to contain six phases: M_1_, M_2_, M_3_, O_1_, O_2_ and T. The M_1_, M_2_ and M_3_ phases have orthorhombic structures (the M label denotes the monoclinic distortion of the primitive unit cell) and all of them exhibit antiferroelectricity (Ratuszna *et al.*, 2003 ▸; Kania, 2001 ▸). The peaks at the ∼*T*_I_ and *T*_II_ points are assigned to the M_1_–M_2_ and M_2_–M_3_ phase transitions, respectively. The sharp peak at the *T*_III_ point is attributed to the phase transition between the AFE M_3_ phase and a paraelectric O_1_ phase with a space-group symmetry of *Cmcm*. Previously, the average structures of the M_1_, M_2_ and M_3_ phases were all assigned to the same *Pbcm* space-group symmetry. The phase transitions between the three phases were interpreted as cation displacements (Levin *et al.*, 2009 ▸, 2010 ▸; Krayzman & Levin, 2010 ▸). The broad *T*_II_ peak was proved to be the result of Nb^5+^ displacement dynamics, while the origin of the frequency-dependent *T*_I_ peak is still under debate (Levin *et al.*, 2009 ▸). Recently, the *T*_I_-related peak was explained as being due to the disappearance of weak ferroelectricity within the recently proposed polar *Pmc*2_1_ phase, *i.e.* the softening of the above-mentioned Γ4− mode (Manish *et al.*, 2015 ▸).

The temperature-dependent dielectric spectra of ANLT3 and ANLT4.5 [Figs. 9 ▸(*b*) and 9 ▸(*c*)] are quite similar to pure AN, again accompanied by three dielectric peaks at *T*_I_, *T*_II_ and *T*_III_. Interestingly, the *T*_I_ peak shifts gradually towards low temperature with increasing concentration of LiTaO_3_ and, at the same time, the refined Γ4− mode at room temperature becomes unstable. Furthermore, the increase in LiTaO_3_ content also shifts the *T*_II_ peak to lower temperature with a larger dielectric constant. As mentioned above, the M_3_–M_2_ phase transition has been associated with differing degrees of order for the Nb ions. Referring to the results of Levin *et al.* (2009 ▸), ordered octahedral tilting will promote the long-range order of the Nb displacements. From our experimental data, the H2 mode drops slightly with increasing *x* (*x* ≤ 0.045), which possibly suggests that the octahedral tilting becomes disordered. Therefore, this order–disorder transition can be activated at a lower thermal energy with increasing dopant concentration, thereby moving the transition point towards lower temperature. With further increases in *x*, an additional dielectric peak *T*_U_ is first observed around 140°C in ANLT5.3 and becomes dominant in the ANLT6 and ANLT9 spectra. After the appearance of the *T*_U_ peak, the *T*_I_, *T*_II_ and *T*_III_ related dielectric peaks become systematically more blurred with increasing *x* and almost unobservable in the dielectric constant spectra of ANLT9, although there are still traces in the dielectric loss spectra. The appearance of the *T*_U_ peak is quite consistent with the results in the Li-doped AgNbO_3_ material system and this dielectric anomaly is clearly related to the phase transition between the *R*3*c* FE and AFE phases (Fu *et al.*, 2011*b* ▸). Therefore, the variation in the *T*_U_ peak as a function of *x* can be well explained by the growth in the phase fraction of the *R*3*c* phase in the ANLT100*x* material system.

**Figure 10:**
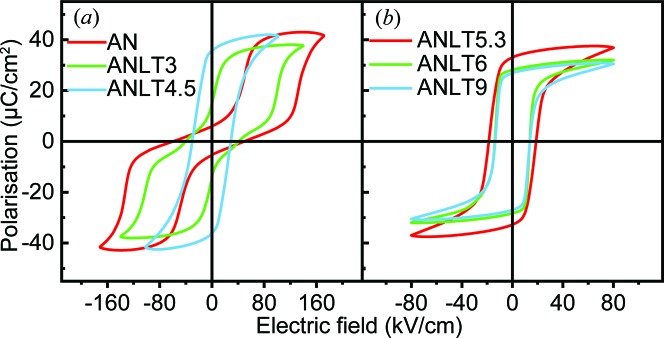
Room-temperature *P*-*E* hysteresis loops for (*a*) AN, ANLT3 and ANLT4.5, and (*b*) ANLT5.3, ANLT6 and ANLT9 bulk ceramics measured at 1 Hz.

Fig. 10 ▸ shows the polarization–electric field (*P*-*E*) hysteresis loops of ANLT100*x* bulk ceramics. Pure AN presents a double *P*-*E* hysteresis loop with an induced polarization of 41 µC cm^−2^ under an applied field of 175 kV cm^−1^. The critical *E* field (*E*_F_) to induce the FE state at 1 Hz is around 125 kV cm^−^ and the non-zero remnant polarization (*P*_r_) is around 6 µC cm^−2^ after withdrawal of the *E* field. These results are almost identical to those reported earlier by Fu *et al.* (2007 ▸). The observed *P*-*E* hysteresis loop confirms the AFE nature of AN, accompanied by weak ferroelectricity. Similar to pure AN, a double *P*-*E* hysteresis loop is also obtained for ANLT3 [Fig. 10 ▸(*a*)] but *E*_F_ decreases to 100 kV cm^−1^ and non-zero *P*_r_ to ∼14 µC cm^−2^. The decrease in the critical field indicates the decreasing energy barrier between the AFE and the induced FE state with increasing *x*. This is consistent with the gradual decrease in the mode amplitude of the Λ3 mode as a function of *x*. The AFE feature, *i.e.* the double *P*-*E* hysteresis loop, disappears experimentally at a composition of ANLT4.5. Instead, a highly saturated single hysteresis loop is observed with a maximum polarization (*P*_m_) ≃ 42 µC cm^−2^ and a *P*_r_ ≃ 36 µC cm^−2^ when a cycled *E* field of 100 kV cm^−1^ is applied. With a further increase in *x*, the ANLT100*x* samples exhibit typical FE features and both *P*_m_ and *P*_r_ decrease slightly. For samples showing two-phase coexistence (*x* ≥ 0.053), the FE properties seem to be determined by the Γ4− mode in the *R*3*c* phase. Intriguingly, with a further increase in the nominal *x* value, the amplitude of the Γ4− mode, *i.e.* the spontaneous polarization, decreases.

**Figure 11:**
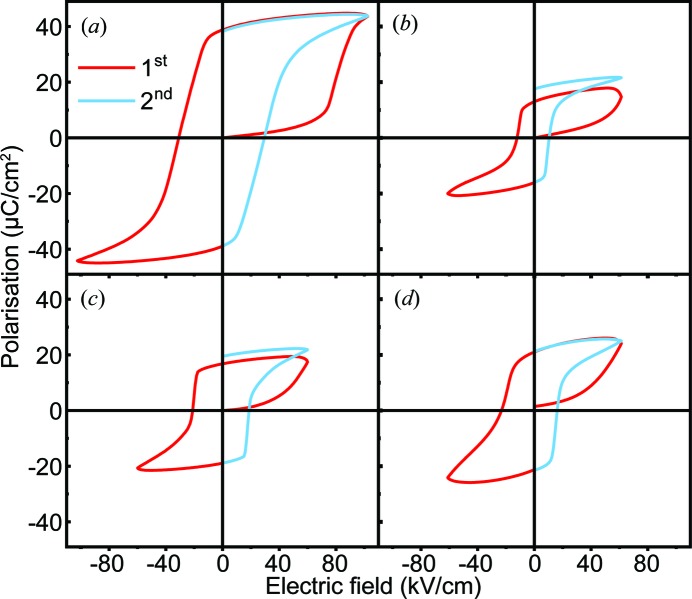
*P*-*E* loops for (*a*) ANLT4.5, (*b*) ANLT5.3, (*c*) ANLT6 and (*d*) ANLT9 measured at 1 Hz in the first cycle (red) and second semi-cycle (blue).

The NPD pattern and temperature-dependent dielectric spectrum of ANLT4.5 suggest that the pristine sample contains a single AFE phase, but its *P*-*E* hysteresis loop shows an FE nature [Fig. 10 ▸(*a*)]. In order to understand the AFE/FE behaviour observed in the ANLT100*x* system, the *P*-*E* hysteresis loops measured in the first and second cycles are displayed in Fig. 11 ▸. It is evident that the polarization of ANLT4.5 increases abruptly after the first quarter *E* field cycle, and after that the *P*-*E* loop behaves like that observed in a classical FE material. This is very similar to the irreversible *E* field-induced AFE–FE phase transition observed in PbZrO_3_-based AFE materials (Lu *et al.*, 2017 ▸; Guo & Tan, 2015 ▸). Furthermore, the *E*_F_ for ANLT4.5 is around 90 kV cm^−1^, presenting a further decrease compared with that observed for ANLT3. For *x* ≥ 0.053, the steep increase in the polarization is hardly observed, and instead the polarization rises gradually over the first quarter cycle. After the first quarter cycle (same amplitude), the *P*_r_ values of ANLT5.3, ANLT6 and ANLT9 are around 13, 17 and 20 µC cm^−2^, respectively. Furthermore, after the second cycle, the *P*_r_ values of ANLT5.3 and ANLT6 show a slight increase, while that of ANLT9 remains almost constant.

**Figure 12:**
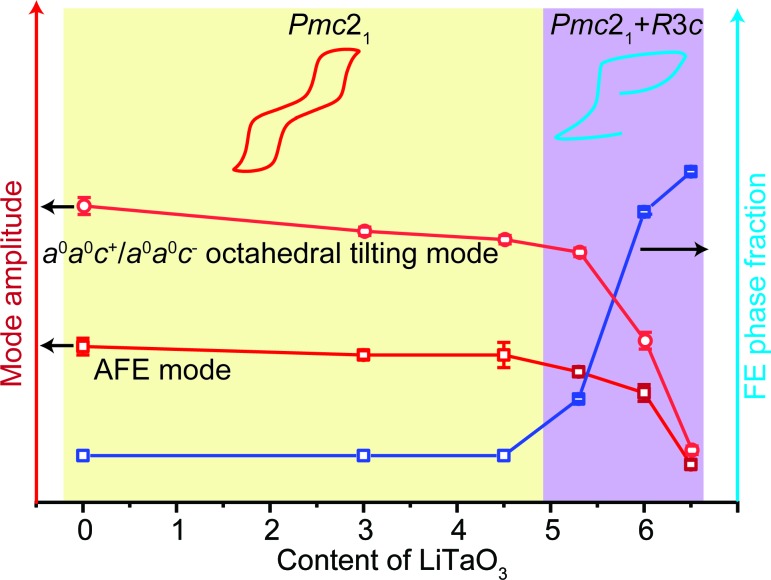
A schematic drawing that reflects the structure–property relationships present in the ANLT100*x* material system in the form of symmetry modes, phases and electrical properties.

The evolution of the measured electrical properties in the ANLT100*x* series can be well understood from a structural viewpoint and is summarized in Fig. 12 ▸. For the pure AN sample, the distorted structure is dominated by two octahedral tilting modes (T4+ and H2), but the secondary Λ3 AFE mode still has a relatively large mode amplitude. Thus, a characteristic double *P*-*E* hysteresis loop is obtained. Upon increasing the content of LiTaO_3_, the H2 mode, *i.e.* the *a* ^0^ *a* ^0^ *c* ^−^/*a* ^0^ *a* ^0^ *c* ^+^ octahedral tilting mode, becomes destabilized due to the heavily underbonded Li^+^ ions and the associated AFE mode is simultaneously damped. The antiferroelectricity hence becomes weak and a lower *E* field is able to trigger an AFE–FE phase transition. Upon further increasing *x* to 0.053, the rhombohedral *R*3*c* phase appears and, at this stage, the samples contain both *Pmc*2_1_ and *R*3*c* phases. The coexistence of the two phases implies a relatively flat energy landscape connecting the *Pmc*2_1_ and *R*3*c* structures. Therefore, as shown by the different *P*-*E* behaviour measured in the first and second cycles, although the virgin state of the sample has an AFE nature, the FE state will be stabilized after applying an *E* field. As the *R*3*c* phase fraction increases, the samples’ antiferroelectricity is further weakened. After applying a first-cycle *E* field with the same amplitude, ANLT9 exhibits the largest remnant polarization.

## 3. Conclusions   

A symmetry-mode decomposition of AgNbO_3_ identifies the differences between the *Pbcm* and *Pmc*2_1_ structures. Both distorted structures share three main modes (T4+, H2 and Λ3) with large amplitudes. The only difference between the *Pbcm* and *Pmc*2_1_ structures is the ‘softening’ of the zone-centre Γ4− mode, which lowers the non-polar *Pbcm* symmetry structure into *Pmc*2_1_ symmetry and is regarded as the origin of the weak ferroelectricity observed in AgNbO_3_. Upon doping LiTaO_3_ into AN, the H2 mode associated with in- or antiphase octahedral tilting around [001]_p_ gradually becomes destabilized, while another octahedral rotation mode (T4+, *a* ^−^ *a* ^−^ *c* ^0^ tilting) shows no such significant variation. The secondary Λ3 mode (induced by the two octahedral rotation modes) controls the antiferroelectric behaviour observed in the samples, which is then damped as a result of the H2 mode destabilization. With further LiTaO_3_ doping, another *R*3*c* phase appears and the samples contain two phases, suggesting a low energy barrier between the *Pmc*2_1_ and *R*3*c* structures. Considering the octahedral tilting modes in both phases, we postulate that the disappearance of the H2 mode as a function of increasing *x* is the main force driving the structural evolution of the ANLT100*x* material system. More importantly, through symmetry-mode analysis this work provides a detailed physical picture of the phase transition in the ANLT100*x* system and builds an intuitive connection to the obtained electrical properties. We believe that this work provides insight into how to tune the electrical properties by controlling the amplitudes of the relative modes in these antiferroelectric and ferroelectric materials. It also introduces a novel approach to structure refinement that provides information on the hidden structural correlations for a better understanding of the materials’ physical properties.

## Supplementary Material

Tables of refinement results. DOI: 10.1107/S2052252519007711/ed5018sup1.pdf

## Figures and Tables

**Table 1:** The dimensions and global amplitudes of distortive modes observed in the *Pmca* and *Pmc*2_1_ structures The **q** vector basis refers to the *Ammm* and pseudo-cubic perovskite structures.

Wavevector **q**				Dimension		*A*_τ_ (Å)	
*Ammm*	Pseudo-cubic	**q**_*i*_	Irrep	*Pmca*	*Pmc*2_1_	*Pmca*	*Pmc*2_1_
[0 0 0]*	[0 0 0]_p_*	**q**_0_	Γ4−		5		0.21
[1/4 0 0]*	[0 0 1/4]_p_*	**q**_1_	Λ1		4		0.09
[1/4 0 0]*	[0 0 1/4]_p_*	**q**_2_	Λ3	5	5	0.48	0.47
[0 1 0]*	[1/2 1/2 0]_p_*	**q**_3_	Y2+		2		0.17
[0 1 0]*	[1/2 1/2 0]_p_*	**q**_4_	Y3−	3	3	0.16	0.16
[1/2 0 0]*	[0 0 1/2]_p_*	**q**_5_	Z3+		2		0.03
[1/2 0 0]*	[0 0 1/2]_p_*	**q**_6_	Z2−	2	2	0.04	0.02
[1/2 1 0]*	[1/2 1/2 1/2]_p_*	**q**_7_	T4+	3	3	1.23	1.22
[1/4 1 0]*	[1/2 1/2 1/4]_p_*	**q**_8_	H2	2	2	1.00	0.97
[1/4 1 0]*	[1/2 1/2 1/4]_p_*	**q**_9_	H4		4		0.11

## References

[bb1] Alonso, J. A., Sanz, J., Santamaría, J., León, C., Várez, A. & Fernández-Díaz, M. T. (2000). *Angew. Chem. Int. Ed.* **39**, 619–621. 10.1002/(sici)1521-3773(20000204)39:3<619::aid-anie619>3.0.co;2-o 10671277

[bb2] Bellaiche, L. & Íñiguez, J. (2013). *Phys. Rev. B*, **88**, 014104.

[bb3] Bellaiche, L. & Vanderbilt, D. (1999). *Phys. Rev. Lett.* **83**, 1347–1350.

[bb4] Brant, W. R., Schmid, S., Kuhn, A., Hester, J., Avdeev, M., Sale, M. & Gu, Q. (2012). *ChemPhysChem*, **13**, 2293–2296. 10.1002/cphc.201200017 22573574

[bb5] Brown, I. D. (1981). *Structure and Bonding in Crystals*, Vol. 2, edited by M. O’Keeffe & A. Navrotsky, pp. 1–13. New York: Academic Press.

[bb6] Campbell, B. J., Stokes, H. T., Tanner, D. E. & Hatch, D. M. (2006). *J. Appl. Cryst.* **39**, 607–614.

[bb7] Damjanovic, D. (1998). *Rep. Prog. Phys.* **61**, 1267–1324.

[bb8] Dove, M. T. (1997). *Am. Mineral.* **82**, 213–244.

[bb9] Faik, A., Orobengoa, D., Iturbe-Zabalo, E. & Igartua, J. M. (2012). *J. Solid State Chem.* **192**, 273–283.

[bb10] Fu, D., Arioka, T., Taniguchi, H., Taniyama, T. & Itoh, M. (2011*a*). *Appl. Phys. Lett.* **99**, 012904.

[bb11] Fu, D., Endo, M., Taniguchi, H., Taniyama, T. & Itoh, M. (2007). *Appl. Phys. Lett.* **90**, 252907.

[bb12] Fu, D., Endo, M., Taniguchi, H., Taniyama, T., Itoh, M. & Koshihara, S. (2011*b*). *J. Phys. Condens. Matter*, **23**, 075901. 10.1088/0953-8984/23/7/075901 21411887

[bb13] Fu, D., Endo, M., Taniguchi, H., Taniyama, T., Koshihara, S. & Itoh, M. (2008). *Appl. Phys. Lett.* **92**, 172905.

[bb14] Fu, D., Itoh, M. & Koshihara, S. (2009). *J. Appl. Phys.* **106**, 104104.

[bb15] Glazer, A. M. (1975). *Acta Cryst.* A**31**, 756–762.

[bb16] Gómez-Pérez, A., Hoelzel, M., Muñoz-Noval, A., García-Alvarado, F. & Amador, U. (2016). *Inorg. Chem.* **55**, 12766–12774. 10.1021/acs.inorgchem.6b02066 27989167

[bb17] Guo, H. & Tan, X. (2015). *Phys. Rev. B*, **91**, 144104.

[bb18] Guo, R., Cross, L. E., Park, S. E., Noheda, B., Cox, D. E. & Shirane, G. (2000). *Phys. Rev. Lett.* **84**, 5423–5426. 10.1103/PhysRevLett.84.5423 10990959

[bb19] Guo, Y., Liu, Y., Withers, R. L., Brink, F. & Chen, H. (2011). *Chem. Mater.* **23**, 219–228.

[bb20] Haertling, G. H. (1999). *J. Am. Ceram. Soc.* **82**, 797–818.

[bb21] Hao, X., Zhai, J. & Yao, X. (2009). *J. Am. Ceram. Soc.* **92**, 1133–1135.

[bb22] Kania, A. (2001). *J. Phys. D Appl. Phys.* **34**, 1447–1455.

[bb23] Khalyavin, D. D., Salak, A. N., Olekhnovich, N. M., Pushkarev, A. V., Radyush, Y. V., Manuel, P., Raevski, I. P., Zheludkevich, M. L. & Ferreira, M. G. S. (2014). *Phys. Rev. B*, **89**, 174414.

[bb24] Khan, H. U., Sterianou, I., Han, Y., Pokorny, J. & Reaney, I. M. (2010). *J. Appl. Phys.* **108**, 064117.

[bb25] Khan, H. U., Sterianou, I., Miao, S., Pokorny, J. & Reaney, I. M. (2012). *J. Appl. Phys.* **111**, 024107.

[bb26] Krayzman, V. & Levin, I. (2010). *J. Phys. Condens. Matter*, **22**, 404201. 10.1088/0953-8984/22/40/404201 21386562

[bb27] Levin, I., Krayzman, V., Woicik, J. C., Karapetrova, J., Proffen, T., Tucker, M. G. & Reaney, I. M. (2009). *Phys. Rev. B*, **79**, 104113.

[bb28] Levin, I., Woicik, J. C., Llobet, A., Tucker, M. G., Krayzman, V., Pokorny, J. & Reaney, I. M. (2010). *Chem. Mater.* **22**, 4987–4995.

[bb29] Liu, Y., Norén, L., Studer, A. J., Withers, R. L., Guo, Y., Li, Y., Yang, H. & Wang, J. (2012). *J. Solid State Chem.* **187**, 309–315.

[bb30] Liu, Z., Chen, X., Peng, W., Xu, C., Dong, X., Cao, F. & Wang, G. (2015). *Appl. Phys. Lett.* **106**, 262901.

[bb31] Lu, T., Studer, A. J., Yu, D., Withers, R. L., Feng, Y., Chen, H., Islam, S. S., Xu, Z. & Liu, Y. (2017). *Phys. Rev. B*, **96**, 214108.

[bb32] Manish, K. N., Prasad, K. G., Saket, A., Rayaprol, S. & Siruguri, V. (2015). *J. Phys. D Appl. Phys.* **48**, 215303.

[bb33] Mirshekarloo, M. S., Yao, K. & Sritharan, T. (2010). *Appl. Phys. Lett.* **97**, 142902.

[bb34] Park, S.-E. & Shrout, T. R. (1997). *J. Appl. Phys.* **82**, 1804–1811.

[bb35] Perez-Mato, J. M., Orobengoa, D. & Aroyo, M. I. (2010). *Acta Cryst.* A**66**, 558–590. 10.1107/S0108767310016247 20720321

[bb36] Prosandeev, S., Wang, D., Ren, W., Íñiguez, J. & Bellaiche, L. (2013). *Adv. Funct. Mater.* **23**, 234–240.

[bb37] Ratuszna, A., Pawluk, J. & Kania, A. (2003). *Phase Transit.* **76**, 611–620.

[bb38] Rodríguez-Carvajal, J. (1993). *Physica B*, **192**, 55–69.

[bb39] Saito, Y., Takao, H., Tani, T., Nonoyama, T., Takatori, K., Homma, T., Nagaya, T. & Nakamura, M. (2004). *Nature*, **432**, 84–87. 10.1038/nature03028 15516921

[bb40] Sciau, P., Kania, A., Dkhil, B., Suard, E. & Ratuszna, A. (2004). *J. Phys. Condens. Matter*, **16**, 2795–2810.

[bb41] Setter, N., Damjanovic, D., Eng, L., Fox, G., Gevorgian, S., Hong, S., Kingon, A., Kohlstedt, H., Park, N. Y., Stephenson, G. B., Stolitchnov, I., Taganstev, A. K., Taylor, D. V., Yamada, T. & Streiffer, S. (2006). *J. Appl. Phys.* **100**, 051606.

[bb42] Shannon, R. D. (1976). *Acta Cryst.* A**32**, 751–767.

[bb43] Shrout, T. R. & Zhang, S. J. (2007). *J. Electroceram.* **19**, 113–126.

[bb44] Stokes, H. T., Hatch, D. M. & Wells, J. D. (1991). *Phys. Rev. B*, **43**, 11010–11018. 10.1103/physrevb.43.11010 9996834

[bb45] Tian, Y., Jin, L., Zhang, H., Xu, Z., Wei, X., Politova, E. D., Stefanovich, S. Y., Tarakina, N. V., Abrahams, I. & Yan, H. (2016). *J. Mater. Chem. A*, **4**, 17279–17287.

[bb46] Tian, Y., Jin, L., Zhang, H., Xu, Z., Wei, X., Viola, G., Abrahams, I. & Yan, H. (2017). *J. Mater. Chem. A*, **5**, 17525–17531.

[bb47] Wang, J., Liu, Y., Withers, R. L., Studer, A., Li, Q., Norén, L. & Guo, Y. (2011). *J. Appl. Phys.* **110**, 084114.

[bb48] Yashima, M., Matsuyama, S., Sano, R., Itoh, M., Tsuda, K. & Fu, D. S. (2011). *Chem. Mater.* **23**, 1643–1645.

[bb49] Zhao, L., Gao, J., Liu, Q., Zhang, S. & Li, J.-F. (2018). *ACS Appl. Mater. Interfaces*, **10**, 819–826. 10.1021/acsami.7b17382 29243905

[bb50] Zhao, L., Liu, Q., Gao, J., Zhang, S. & Li, J. (2017). *Adv. Mater.* **29**, 1701824. 10.1002/adma.201701824 28628242

[bb51] Zhao, L., Liu, Q., Zhang, S. & Li, J.-F. (2016). *J. Mater. Chem. C.* **4**, 8380–8384.

