# Supplementary material for: Symmetry-mode analysis for intuitive observation of structure–property relationships in the lead-free antiferroelectric (1−x)AgNbO3–xLiTaO3
Source: IUCrJ. 2019 Jun 21;6(Pt 4):740–50. doi: 10.1107/S2052252519007711 (PMC6608632; doi:10.1107/S2052252519007711)
Supplement: Supplementary file 1 [file m-06-00740-sup1.pdf]

IUCrJ

Volume 6 (2019)

Supporting information for article:

Symmetry-mode analysis for intuitive observation of structure–property relationships in the lead-free antiferroelectric (1−*x*)AgNbO3–*x*LiTaO3

Teng Lu, Ye Tian, Andrew Studer, Narendirakumar Narayanan, Qian Li, Ray Withers, Li Jin, Y. Mendez-González, A. Peláiz-Barranco, Dehong Yu, Garry J. McIntye, Zhuo Xu, Xiaoyong Wei, Haixue Yan and Yun Liu

S1. The symmetry-mode decomposition of the *Pmca* and *Pmc*2_1 structures.

Table S1 The symmetry-mode decomposition results for the *Pmca* structure referring to the *Ammm* parent structure. The coordinates after the atomic label are those for the undistorted structure. The unit-cell parameters for the *Pmca* structure are: *a*=15.6503 Å, *b*=5.5517 Å, *c*=5.6080 Å, *α*=*β*=*γ*=90°.

Wave-vector	Irrep	Atomic Label			
		O1	0.75	0.75	0.75
			δx	*δy*	*δz*
**[1/4 0 0]***	Λ3		0	0	0.01361
**[0 1 0]***	Y3-		0	0.00605	0
**[1/2 1 0]***	T4+		0	-0.05526	0
		O2	0.5	0.75	0.75
			δx	*δy*	*δz*
**[0 1 0]***	Y3-		0	0.00605	0
**[1/2 1 0]***	T4+		0	0.05526	0
		O3	0.625	0.5	0.5
			δx	*δy*	*δz*
**[1/4 0 0]***	Λ3		0	0.00085	0.00325
**[1/2 0 0]***	Z2-		-0.00025	0	0
**[1/2 1 0]***	T4+		-0.01387	0	0
**[1/4 1 0]***	H2		0	-0.03193	0.03186
		O4	0.625	0	0.5
			δx	*δy*	*δz*
**[1/4 0 0]***	Λ3		0	-0.00085	0.00325
**[1/2 0 0]***	Z2-		-0.00025	0	0
**[1/2 1 0]***	T4+		0.01387	0	0
**[1/4 1 0]***	H2		0	-0.03193	-0.03186
		Ag1	0.75	0.75	0.25
			δx	*δy*	*δz*
**[1/4 0 0]***	Λ3		0	0	-0.01681
**[0 1 0]***	Y3-		0	0.00705	0
**[1/2 1 0]***	T4+		0	0.00035	0
		Ag2	0.5	0.75	0.25
			δx	*δy*	*δz*
**[0 1 0]***	Y3-		0	0.00705	0
**[1/2 1 0]***	T4+		0	-0.00035	0
		Nb1	0.625	0.75	0.75

			δx	δy	δz
**[1/4 0 0]***	Λ3		0	0	-0.02558
**[0 1 0]***	Y3-		0	-0.0044	0

Table S2 The symmetry-mode decomposition results for the $Pmc2_1$ structure relative to the *Ammm* parent structure. The coordinates after the atomic label are those for the undistorted structure. Note that as the $Pmc2_1$ structure is polar along the *c*-direction, the atomic coordinates exhibit origin shifts along **c**. The unit-cell parameters for $Pmc2_1$ structure are: a=15.6459 Å, b=5.5525 Å, c=5.6091 Å, α=β=γ=90°.

Wave-vector	Irrep	Atomic Label							
		O1	0.75	0.75	0.768	Ag1	0.75	0.75	0.268
			δx	δy	δz		δx	δy	δz
**[0 0 0]***	Γ4-		0	0	-0.00441		0	0	-0.00042
**[1/4 0 0]***	Λ1		0.0004	0	0		0.0004	0	0
**[0 1 0]***	Y3-		0	0.00455	0		0	-0.0042	0
**[1/2 0 0]***	Z3+		0	0	-0.00125		0	0	-0.00125
**[1/2 1 0]***	T4+		0	-0.05497	0		0	0.0013	0
		O2	0.5	0.75	0.768	Ag2	0.5	0.75	0.268
			δx	δy	δz		δx	δy	δz
**[0 0 0]***	Γ4-		0	0	-0.00441		0	0	-0.00042
**[1/4 0 0]***	Λ3		0	0	0.01849		0	0	-0.01949
**[0 1 0]***	Y3-		0	0.00455	0		0	-0.0042	0
**[1/2 0 0]***	Z3+		0	0	0.00125		0	0	0.00125
**[1/2 1 0]***	T4+		0	0.05497	0		0	-0.0013	0
**[1/4 1 0]**	H4		0	0.0095	0		0	0.0015	0
		O3	0.625	0.5	0.518	O4	0.625	0	0.518
			δx	δy	δz		δx	δy	δz
**[0 0 0]***	Γ4-		0	0.00175	-0.00492		0	-0.00175	-0.00492
**[1/4 0 0]***	Λ1		-0.00135	0	0		-0.00135	0	0
**[1/4 0 0]***	Λ3		0	0.00025	0.00275		0	-0.00025	0.00275
**[0 1 0]***	Y2+		0	0.00575	-0.00525		0	0.00575	0.00525
**[1/2 0 0]***	Z2-		0.0002	0	0		0.0002	0	0
**[1/2 1 0]***	T4+		-0.01392	0	0		0.01392	0	0
**[1/4 1 0]***	H2		0	0.03123	-0.03027		0	0.03123	0.03027
**[1/4 1 0]***	H4		-0.00055	0	0		0.00055	0	0
		O5	0	0.25	0.268	Ag3	0	0.75	0.268
			δx	δy	δz		δx	δy	δz

**[0 0 0]***	Γ4-		0	0	-0.00441		0	0	-0.00042
**[1/4 0 0]***	Λ3		0	0	-0.01849		0	0	0.01949
**[0 1 0]***	Y3-		0	-0.00455	0		0	-0.0042	0
**[1/2 0 0]***	Z3+		0	0	0.00125		0	0	0.00125
**[1/2 1 0]***	T4+		0	-0.05497	0		0	-0.0013	0
**[1/4 1 0]**	H4		0	0.0095	0		0	-0.0015	0
		O6	0.125	0	0.018	O7	0.125	0.5	0.518
			δx	*δy*	*δz*		*δx*	*δy*	*δz*
**[0 0 0]***	Γ4-		0	0.00175	-0.00492		0	0.00175	-0.00492
**[1/4 0 0]***	Λ1		0.00135	0	0		0.00135	0	0
**[1/4 0 0]***	Λ3		0	-0.00025	-0.00275		0	-0.00025	-0.00275
**[0 1 0]***	Y2+		0	-0.00575	0.00525		0	0.00575	-0.00525
**[1/2 0 0]***	Z2-		0.0002	0	0		0.0002	0	0
**[1/2 1 0]***	T4+		0.01392	0	0		-0.01392	0	0
**[1/4 1 0]***	H2		0	0.03123	-0.03027		0	-0.03123	0.03027
**[1/4 1 0]***	H4		-0.00055	0	0		-0.00055	0	0
		Nb1	0.625	0.75	0.768	Nb2	0.125	0.25	0.268
			δx	*δy*	*δz*		*δx*	*δy*	*δz*
**[0 0 0]***	Γ4-		0	0	-0.01046		0	0	-0.01046
**[1/4 0 0]***	Λ1		0.0001	0	0		-0.0001	0	0
**[1/4 0 0]***	Λ3		0	0	-0.02259		0	0	0.02259
**[0 1 0]***	Y3-		0	0.00776	0		0	-0.00776	0
**[1/2 0 0]***	Z2-		0.0005	0	0		0.0005	0	0
**[1/4 1 0]**	H4		0	-0.00066	0		0	-0.00066	0

S2. Bond valence sum calculations on $AgNbO_3$ and $LiTaO_3$

Bond valence calculations using conventional BV parameters: $AgNbO_3$ $Pmc2_1$ space group symmetry; a = 15.6542 Å; b = 5.5464 Å, c = 5.6015 Å.

Ag3-O5: 2.8867Å s=0.0594v.u. ; 2.6833Å s=0.1029v.u. ; 2.5666Å s=0.1411v.u. ; 3.0739Å s=0.0358v.u.
Ag3-O6: 2.7756Å s=0.0802v.u. ; 2.7756Å s=0.0802v.u. ; 3.2319Å s=0.0234v.u. ; 3.2319Å s=0.0234v.u.
Ag3-O7: 2.6635Å s=0.1086v.u. ; 2.5475Å s=0.1486v.u. ; 2.6635Å s=0.1086v.u. ; 2.5475Å s=0.1486v.u.

BV sum of Ag3: 1.0608v.u. ; BV sum deviation: 0.0608v.u.

Ag1-O1: 2.8492Å s=0.0657v.u. ; 2.7866Å s=0.0779v.u. ; 2.4125Å s=0.2140v.u. 3.1343Å s=0.0304v.u.
Ag1-O6: 2.4489Å s=0.1939v.u. ; 2.8339Å s=0.0685v.u.
Ag1-O7: 2.7741Å s=0.0805v.u. ; 3.0003Å s=0.0437v.u.
Ag1-O4: 2.8590Å s=0.0640v.u. ; 2.4426Å s=0.1973v.u.
Ag1-O3: 2.7697Å s=0.0815v.u. ; 3.1595Å s=0.0284v.u.

BV sum of Ag1: 1.1458v.u. ; BV sum deviation: 0.1458v.u.

Ag2-O2: 2.6267Å s=0.1199v.u. ; 3.0089Å s=0.0427v.u. ; 2.4885Å s=0.1742v.u. ; 3.0713Å s=0.0361v.u.
Ag2-O4: 2.7250Å s=0.0920v.u. ; 2.7250Å s=0.0920v.u. ; 3.1798Å s=0.0269v.u. ; 3.1798Å s=0.0269v.u.
Ag2-O3: 2.4273Å s=0.2056v.u. ; 2.7798Å s=0.0793v.u. ; 2.4273Å s=0.2056v.u. ; 2.7798Å s=0.0793v.u.

BV sum of Ag2: 1.1804v.u. ; BV sum deviation: 0.1804v.u.

Nb2-O5: 1.9777Å s=0.8351v.u.
Nb2-O1: 1.9829Å s=0.8234v.u.
Nb2-O6: 2.0102Å s=0.7648v.u. ; 1.9263Å s=0.9594v.u.
Nb2-O7: 1.9437Å s=0.9155v.u. ; 2.0928Å s=0.6117v.u.

BV sum of Nb2: 4.9100v.u. ; BV sum deviation: -0.0900v.u.

Nb1-O1: 1.9895Å s=0.8088v.u.
Nb1-O2: 2.0120Å s=0.7611v.u.
Nb1-O4: 1.8653Å s=1.1316v.u. ; 2.1852Å s=0.4766v.u.
Nb1-O3: 1.8645Å s=1.1340v.u. ; 2.1263Å s=0.5589v.u.

BV sum of Nb1: 4.8709v.u. ; BV sum deviation: -0.1291v.u.

Ag3-O5: 2.5666Å s=0.1411v.u. ; 2.6833Å s=0.1029v.u. ; 2.8867Å s=0.0594v.u. ; 3.0739Å s=0.0358v.u.
Nb2-O5: 1.9776Å s=0.8352v.u. ; 1.9776Å s=0.8352v.u.

BV sum of O5: 2.0096v.u. ; BV sum deviation: 0.0096v.u.

Ag1-O1: 2.4125Å s=0.2140v.u. 2.8492Å s=0.0657v.u. ; 2.7866Å s=0.0779v.u. ; 3.1343Å s=0.0304v.u.
Nb2-O1: 1.9829Å s=0.8234v.u.
Nb1-O1: 1.9895Å s=0.8089v.u.

BV sum of O1: 2.0202v.u. ; BV sum deviation: 0.0202v.u.

Ag2-O2: 2.4886Å s=0.1742v.u. ; 2.6266Å s=0.1200v.u. ; 3.0089Å s=0.0427v.u. ; 3.0713Å s=0.0361v.u.
Nb1-O2: 2.0120Å s=0.7610v.u. ; 2.0120Å s=0.7610v.u.

BV sum of O2: 2.015v.u. ; BV sum deviation: 0.015v.u.

Ag3-O6: 2.7757Å s=0.0802v.u. ; 3.2319Å s=0.0234v.u.
Ag1-O6: 2.4490Å s=0.1939v.u. ; 2.8339Å s=0.0685v.u.
Nb2-O6: 2.0103Å s=0.7646v.u. ; 1.9263Å s=0.9595v.u.

BV sum of O6: 2.09v.u. ; BV sum deviation: 0.09v.u.

Ag3-O7: 2.6635Å s=0.1086v.u. ; 2.5476Å s=0.1485v.u.
Ag1-O7: 2.7741Å s=0.0805v.u. ; 3.0003Å s=0.0437v.u.
Nb2-O7: 1.9437Å s=0.9155v.u. ; 2.0929Å s=0.6116v.u.

BV sum of O7: 1.9134v.u. ; BV sum deviation: -0.0866v.u.

Ag1-O4: 2.4426Å s=0.1973v.u. ; 2.8590Å s=0.0640v.u.
Ag2-O4: 2.7249Å s=0.0920v.u. ; 3.1798Å s=0.0269v.u.
Nb1-O4: 1.8653Å s=1.1315v.u. ; 2.1852Å s=0.4766v.u.

BV sum of O4: 1.8974v.u. ; BV sum deviation: -0.0117v.u.

Ag1-O3: 2.7697Å s=0.0815v.u. ; 3.1595Å s=0.0284v.u.
Ag2-O3: 2.4274Å s=0.2055v.u. ; 2.7798Å s=0.0793v.u.
Nb1-O3: 1.8645Å s=1.1340v.u. ; 2.1262Å s=0.5591v.u.

BV sum of O3: 2.0878v.u. ; BV sum deviation: 0.0878v.u.

Global instability index GII = 0.0945v.u.

Bond valence calculations using conventional BV parameters: $LiTaO_3$ $Pmc2_1$ space group symmetry; a = 15.6542 Å; b = 5.5464 Å, c = 5.6015 Å.

Li3-O5: 2.8867Å s=0.0215v.u. ; 2.6833Å s=0.0373v.u. ; 2.5666Å s=0.0511v.u. ; 3.0739Å s=0.0130v.u.
Li3-O6: 2.7756Å s=0.0290v.u. ; 2.7756Å s=0.0290v.u. ; 3.2319Å s=0.0085v.u. ; 3.2319Å s=0.0085v.u.
Li3-O7: 2.6635Å s=0.0393v.u. ; 2.5475Å s=0.0538v.u. ; 2.6635Å s=0.0393v.u. ; 2.5475Å s=0.0538v.u.

BV sum of Li3: 0.3841v.u. ; BV sum deviation: -0.6159v.u.

Li1-O1: 2.8492Å s=0.0238v.u. ; 2.7866Å s=0.0282v.u. ; 2.4125Å s=0.0774v.u. 3.1343Å s=0.0110v.u.
Li1-O6: 2.4489Å s=0.0702v.u. ; 2.8339Å s=0.0248v.u.
Li1-O7: 2.7741Å s=0.0292v.u. ; 3.0003Å s=0.0158v.u.
Li1-O4: 2.8590Å s=0.0232v.u. ; 2.4426Å s=0.0714v.u.
Li1-O3: 2.7697Å s=0.0295v.u. ; 3.1595Å s=0.0103v.u.

BV sum of Li1: 0.4148v.u. ; BV sum deviation: -0.5852v.u.

Li2-O2: 2.6267Å s=0.0434v.u. ; 3.0089Å s=0.0155v.u. ; 2.4885Å s=0.0631v.u. ; 3.0713Å s=0.0131v.u.
Li2-O4: 2.7250Å s=0.0333v.u. ; 2.7250Å s=0.0333v.u. ; 3.1798Å s=0.0097v.u. ; 3.1798Å s=0.0097v.u.
Li2-O3: 2.4273Å s=0.0744v.u. ; 2.7798Å s=0.0287v.u. ; 2.4273Å s=0.0744v.u. ; 2.7798Å s=0.0287v.u.

BV sum of Li2: 0.4273v.u. ; BV sum deviation: -0.5727v.u.

Ta2-O5: 1.9777Å s=0.8557v.u.
Ta2-O1: 1.9829Å s=0.8437v.u.
Ta2-O6: 2.0102Å s=0.7836v.u. ; 1.9263Å s=0.9831v.u.
Ta2-O7: 1.9437Å s=0.9380v.u. ; 2.0928Å s=0.6268v.u.

BV sum of Ta2: 5.0309v.u. ; BV sum deviation: 0.0309v.u.

Ta1-O1: 1.9895Å s=0.8287v.u.
Ta1-O2: 2.0120Å s=0.7798v.u.

Ta1-O4: 1.8653Å s=1.1594v.u. ; 2.1852Å s=0.4884v.u.
Ta1-O3: 1.8645Å s=1.1619v.u. ; 2.1263Å s=0.5726v.u.

BV sum of Ta1: 4.9909v.u. ; BV sum deviation: -0.0091v.u.

Ta2-O5: 1.9776Å s=0.8558v.u. ; 1.9776Å s=0.8558v.u.

Li3-O5: 2.8867Å s=0.0215v.u. ; 2.6833Å s=0.0373v.u. ; 2.5666Å s=0.0511v.u. ; 3.0739Å s=0.0130v.u.

BV sum of O5: 1.7115v.u. ; BV sum deviation: -0.2885v.u.

Ta2-O1: 1.9829Å s=0.8436v.u.
Ta1-O1: 1.9895Å s=0.8288v.u.

Li1-O1: 2.8492Å s=0.0238v.u. ; 2.7866Å s=0.0282v.u. ; 2.4125Å s=0.0774v.u. 3.1343Å s=0.0110v.u.

BV sum of O1: 1.8128v.u. ; BV sum deviation: -0.1872v.u.

Ta1-O2: 2.0120Å s=0.7798v.u. ; 2.0120Å s=0.7798v.u.

Li2-O2: 2.6267Å s=0.0434v.u. ; 3.0089Å s=0.0155v.u. ; 2.4885Å s=0.0631v.u. ; 3.0713Å s=0.0131v.u.

BV sum of O2: 1.6947v.u. ; BV sum deviation: -0.3035v.u.

Ta2-O6: 2.0103Å s=0.7834v.u. ; 1.9263Å s=0.9831v.u.

Li3-O6: 2.7756Å s=0.0290v.u. ; 3.2319Å s=0.0085v.u.

Li1-O6: 2.4489Å s=0.0702v.u. ; 2.8339Å s=0.0248v.u.

BV sum of O6: 1.8989v.u. ; BV sum deviation: -0.1011v.u.

Ta2-O7: 1.9437Å s=0.9380v.u. ; 2.0929Å s=0.6267v.u.

Li3-O7: 2.6635Å s=0.0393v.u. ; 2.5475Å s=0.0538v.u.

Li1-O7: 2.7741Å s=0.0292v.u. ; 3.0003Å s=0.0158v.u.

BV sum of O7: 1.7028v.u. ; BV sum deviation: -0.2972v.u.

Ta1-O4: 1.8653Å s=1.1594v.u. ; 2.1852Å s=0.4883v.u.

Li1-O4: 2.8590Å s=0.0232v.u. ; 2.4426Å s=0.0714v.u.

Li2-O4: 2.7250Å s=0.0333v.u. ; 3.1798Å s=0.0097v.u.

BV sum of O4: 1.7853v.u. ; BV sum deviation: -0.2147v.u.

Ta1-O3: 1.8645Å s=1.1619v.u. ; 2.1262Å s=0.5728v.u.

Li1-O3: 2.7697Å s=0.0295v.u. ; 3.1595Å s=0.0103v.u.

Li2-O3: 2.4273Å s=0.0744v.u. ; 2.7798Å s=0.0287v.u.

BV sum of O3: 1.8776v.u. ; BV sum deviation: -0.1224v.u.

Global instability index GII = 0.3794v.u.

S3. Rietveld refinement results from ANLT100*x* (*x*=0, 0.03, 0.045, 0.053, 0.06 and 0.09)

Table S3 The refined crystal lattice parameters and reliability factors of the ANLT100*x* materials resulting from the Rietveld analyses based on the NPD data.

Chemical composition		AN	ANLT3	ANLT4.5	ANLT5.3		ANLT6		ANLT9	
Space group		*Pmc*2$_1$	*Pmc*2$_1$	*Pmc*2$_1$	*Pmc*2$_1$	*R*3*c*	*Pmc*2$_1$	*R*3*c*	*Pmc*2$_1$	*R*3*c*
Unit-cell parameters (Å)	*a*	15.6542(25)	15.6530(25)	15.6473(24)	15.6520(22)	5.5241(8)	15.6775(26)	5.5240(8)	15.6927(25)	5.520(1)
	b	5.5464(8)	5.5402(8)	5.5354(8)	5.5362(8)	5.5241(8)	5.5323(8)	5.5240(8)	5.5254(11)	5.520(1)
	c	5.6015(7)	5.6000(7)	5.5964(7)	5.5989(7)	13.7957(18)	5.5968(7)	13.7860(18)	5.5884(12)	13.7788(22)
R_p		1.88%	1.99%	1.80%	1.87%	1.87%	2.00%	2.00%	2.60%	2.60%
R_{wp}		2.33%	2.50%	2.22%	2.30%	2.30%	2.51%	2.51%	3.35%	3.35%
R_{ex}		1.44%	1.44%	1.45%	1.43%	1.43%	1.45%	1.45%	1.45%	1.45%
R_{Bragg}		2.60%	2.64%	3.55%	2.25%	2.50%	4.54%	3.93%	5.71%	4.15%
R_F		1.89%	1.93%	2.68%	1.89%	1.25%	3.67%	1.77%	5.40%	2.14%

Table S4 Refined crystal structure parameters from the NPD data for $AgNbO_3$ at room temperature.

Space group	*Pmc*2$_1$	Fractional coordinate			Biso(Å^2)
Atomic Label	Wyckoff position	*x*	*y*	*z*	
Ag3	2a	0	0.7295(10)	0.2855(13)	1.108(7)
Ag1	4c	0.7504(9)	0.7454(10)	0.2663(13)	1.108(7)
Ag2	2b	0.5	0.7485(10)	0.2521(13)	1.108(7)
Nb2	4c	0.1254(5)	0.2412(10)	0.2717(11)	0.61(3)
Nb1	4c	0.6256(5)	0.7502(10)	0.7302(11)	0.61(3)
O5	2a	0	0.2110(14)	0.240(2)	0.93(2)
O1	4c	0.7504(13)	0.6895(14)	0.761(2)	0.93(2)
O2	2b	0.5	0.8021(14)	0.783(2)	0.93(2)
O6	4c	0.1391(3)	0.0309(5)	-0.0192(18)	0.93(2)
O7	4c	0.1144(3)	0.4721(5)	0.5297(18)	0.93(2)
O4	4c	0.6391(3)	0.0327(5)	0.5521(18)	0.93(2)
O3	4c	0.6086(3)	0.5367(5)	0.4760(18)	0.93(2)

Table S5 Refined crystal structure parameters from the NPD data for 97%$AgNbO_3$-3%$LiTaO_3$ at room temperature.

Space group	*Pmc*2₁	Fractional coordinate			Biso(Å²)
Atomic label	Wyckoff position	*x*	*y*	*z*	
Ag3/Li3	2a	0	0.733(2)	0.284(2)	1.09(5)
Ag1/Li1	4c	0.7533(9)	0.750(2)	0.2651(18)	1.09(5)
Ag2/Li2	2b	0.5	0.751(2)	0.256(2)	1.09(5)
Nb2/Ta2	4c	0.1244(8)	0.2394(10)	0.2722(15)	0.56(3)
Nb1/Ta1	4c	0.6277(8)	0.7537(10)	0.7303(15)	0.56(3)
O5	2a	0	0.1909(17)	0.235(3)	1.05(2)
O1	4c	0.7505(10)	0.6992(17)	0.752(3)	1.05(2)
O2	2b	0.5	0.8055(17)	0.776(2)	1.05(2)
O6	4c	0.1401(7)	0.02725	-0.014(2)	1.05(2)
O7	4c	0.1140(7)	0.47881	0.528(2)	1.05(2)
O4	4c	0.6391(7)	0.03597	0.554(2)	1.05(2)
O3	4c	0.6080(7)	0.53691	0.478(2)	1.05(2)

Table S6 Refined crystal parameters of NPD data for 95.5%$AgNbO_3$-4.5%$LiTaO_3$ at room temperature.

Space group	*Pmc*2₁	Fractional coordinate			Biso(Å²)
Atomic label	Wyckoff position	*x*	*y*	*z*	
Ag3/Li3	2a	0	0.732(2)	0.283(1)	0.92(3)
Ag1/Li1	4c	0.752(1)	0.752(2)	0.2644(16)	0.92(3)
Ag2/Li2	2b	0.5	0.758(2)	0.258(2)	0.92(3)
Nb2/Ta2	4c	0.1260(8)	0.2382(11)	0.2686(17)	0.45(2)
Nb1/Ta1	4c	0.6282(9)	0.7529(11)	0.7288(17)	0.45(2)
O5	2a	0	0.1902(19)	0.226(3)	0.974(18)
O1	4c	0.7509(11)	0.6949(19)	0.74979	0.974(18)
O2	2b	0.5	0.8118(19)	0.774(3)	0.974(18)
O6	4c	0.1405(9)	0.0225(18)	-0.010(2)	0.974(18)
O7	4c	0.1152(9)	0.4773(18)	0.529(2)	0.974(18)
O4	4c	0.6410(9)	0.0390(18)	0.556(2)	0.974(18)
O3	4c	0.6087(9)	0.5341(18)	0.480(2)	0.974(18)

Table S7 Refined crystal structure parameters from the NPD data for 94.7%$AgNbO_3$-5.3%$LiTaO_3$ at room temperature.

Space group	*Pmc*2$_1$	Fractional coordinate			Biso(Å^2)
Atomic label	Wyckoff position	*x*	*y*	*z*	
Ag3/Li3	2a	0	0.739(2)	0.2836(12)	1.00(4)
Ag1/Li1	4c	0.7545(18)	0.762(2)	0.264(1)	1.00(4)
Ag2/Li2	2b	0.5	0.7614(9)	0.2590(12)	1.00(4)
Nb2/Ta2	4c	0.1256(3)	0.2458(12)	0.2666(12)	0.81(2)
Nb1/Ta1	4c	0.6283(3)	0.7461(11)	0.7253(12)	0.81(2)
O5	2a	0	0.1883(6)	0.230(3)	1.254(18)
O1	4c	0.7482(10)	0.7033(6)	0.748(3)	1.254(18)
O2	2b	0.5	0.8272(6)	0.765(3)	1.254(18)
O6	4c	0.1380(3)	0.0202(6)	-0.017(3)	1.254(18)
O7	4c	0.1113(3)	0.4801(6)	0.525(3)	1.254(18)
O4	4c	0.6385(3)	0.0394(6)	0.543(3)	1.254(18)
O3	4c	0.6086(3)	0.5341(6)	0.472(3)	1.254(18)
Space group	*R*3*c*	Fractional coordinate			Biso(Å^2)
Atomic label	Wyckoff position	*x*	*y*	*z*	
Ag1/Li1	6a	0	0	0.3253	1.00(4)
Nb1/Ta1	6a	0	0	0.0838(10)	0.81(2)
O1	18b	0.5726(17)	1.0265(19)	0.3163(8)	1.254(18)

Table S8 Refined crystal structure parameters from the NPD data for 94%$AgNbO_3$-6%$LiTaO_3$ at room temperature.

Space group	*Pmc*2$_1$	Fractional coordinate			Biso(Å^2)
Atomic label	Wyckoff position	*x*	*y*	*z*	
Ag3/Li3	2a	0	0.741(3)	0.2899(18)	1.02(3)
Ag1/Li1	4c	0.750(2)	0.746(3)	0.2542(18)	1.02(3)
Ag2/Li2	2b	0.5	0.756(3)	0.2721(18)	1.02(3)
Nb2/Ta2	4c	0.1233(18)	0.245(2)	0.251(3)	0.62(2)
Nb1/Ta1	4c	0.6235(18)	0.747(2)	0.728(3)	0.62(2)
O5	2a	0	0.225(2)	0.243(3)	1.1490(6)
O1	4c	0.7504(10)	0.688(2)	0.748(2)	1.1490(6)
O2	2b	0.5	0.790(2)	0.772(2)	1.1490(6)
O6	4c	0.1387(7)	0.0088(15)	-0.002(3)	1.1490(6)
O7	4c	0.1096(7)	0.4833(15)	0.523(3)	1.1490(6)
O4	4c	0.6387(7)	0.0226(15)	0.534(3)	1.1490(6)
O3	4c	0.6042(7)	0.5258(15)	0.473(3)	1.1490(6)
Space group	*R*3*c*	Fractional coordinate			Biso(Å^2)
Atomic label	Wyckoff position	*x*	*y*	*z*	

Ag1/Li1	6a	0	0	0.3253	1.02(3)
Nb1/Ta1	6a	0	0	0.0811(6)	0.62(2)
O1	18b	0.5659(10)	1.0086(9)	0.3175(5)	1.1490(6)

Table S9 Refined crystal structure parameters from the NPD data for 91%$AgNbO_3$-9%$LiTaO_3$ at room temperature.

Space group	*Pmc*2$_1$	Fractional coordinate			Biso(Å^2)
Atomic label	Wyckoff position	*x*	*y*	*z*	
Ag3/Li3	2a	0	0.754(3)	0.251(3)	0.77(6)
Ag1/Li1	4c	0.749(2)	0.749(3)	0.287(2)	0.77(6)
Ag2/Li2	2b	0.5	0.769(3)	0.245(2)	0.77(6)
Nb2/Ta2	4c	0.1248(12)	0.243(1)	0.243(3)	0.79(4)
Nb1/Ta1	4c	0.6284(12)	0.749(1)	0.762(3)	0.79(4)
O5	2a	0	0.183(2)	0.230(3)	1.26(3)
O1	4c	0.7505(10)	0.723(2)	0.732(3)	1.26(3)
O2	2b	0.5	0.800(2)	0.736(3)	1.26(3)
O6	4c	0.141(1)	0.023(2)	-0.022(3)	1.26(3)
O7	4c	0.110(1)	0.485(2)	0.528(3)	1.26(3)
O4	4c	0.643(1)	0.0088(17)	0.510(3)	1.26(3)
O3	4c	0.610(1)	0.4963(17)	0.499(3)	1.26(3)
Space group	*R*3*c*	Fractional coordinate			Biso(Å^2)
Atomic label	Wyckoff position	*x*	*y*	*z*	
Ag1/Li1	6a	0	0	0.3253	0.77(6)
Nb1/Ta1	6a	0	0	0.0848(8)	0.79(4)
O1	18b	0.5687(6)	1.010(1)	0.3200(7)	1.26(3)

S4. Experimental Methods

Polycrystalline ceramic samples were synthesized by conventional solid-state reaction. Raw metal oxides, Ag_2O (99.7%), Nb_2O_5 (99.99%), Li_2CO_3 (99.5%) and Ta_2O_5 (99.99%) were weighed according to the nominal formula of $(1-x)AgNbO_3$-$xLiTaO_3$ (ANLT100*x*, where *x*=0, 0.03, 0.045, 0.053, 0.06 and 0.09 mole) and ball-milled in ethanol for 12 hours. After drying, the mixtures were put into alumina porcelain boats and calcined at 850~950 °C for 4 hours in oxygen atmosphere with a heating rate of 5 ^{o}C/min. The resultant powders were ball-milled again for 4 hours in an ethanol medium. After drying, the powders were mixed with 5 wt% polyvinyl alcohol (PVA) solution and pressed into pellets with a diameter of 15 mm and a thickness of 1~2 mm under 400 MPa uniaxial pressure. These pellets were then heat-treated at 600 °C for 2 hours to burn out the PVA, and then finally sintered at

1060~1120°C for 6 hours in an oxygen atmosphere, followed by a cooling process with a speed of 5 ^{o}C/min down to 500 ^{o}C and then natural cooling to room temperature in an oxygen atmosphere. The resultant ceramic pellets are bright yellow. All the samples possess a higher relative density than 97%.

Neutron diffraction patterns were collected at a wavelength of 1.635 Å using WOMBAT, the high-intensity powder diffractometer at the Australian Nuclear Science and Technology Organisation (ANSTO). The neutron diffraction set-up is the same as the previous description. The ANLT100x samples were fixed at the centre of the stage and rotated around the vertical axis by a step size of 15°. In order to minimize the effect of the texture, in total 13 patterns were added together to produce the diffraction patterns presented hereafter.

For electrical property measurements, a silver electrode was deposited on both surfaces of the ceramic pellets. The temperature-dependent dielectric spectra were collected during the heating process by an LCR meter (Agilent E4980A, Agilent Technologies, Penang, Malaysia) that is connected to a computer-controlled temperature chamber. For polarisation-electric field (P-E) hysteresis loop measurements, gold electrodes were sputtered on the two main surfaces of the annealed specimens for 3 minutes. The measurement was carried out using a ferroelectric measurement system (TF Analyzer 2000, Aachen, Germany).
